# A Genetic Relationship between Phosphorus Efficiency and Photosynthetic Traits in Soybean As Revealed by QTL Analysis Using a High-Density Genetic Map

**DOI:** 10.3389/fpls.2016.00924

**Published:** 2016-06-28

**Authors:** Hongyan Li, Yuming Yang, Hengyou Zhang, Shanshan Chu, Xingguo Zhang, Dongmei Yin, Deyue Yu, Dan Zhang

**Affiliations:** ^1^Collaborative Innovation Center of Henan Grain Crops, College of Agronomy, Henan Agricultural UniversityZhengzhou, China; ^2^National Key Laboratory of Crop Genetics and Germplasm Enhancement, National Center for Soybean Improvement, Nanjing Agricultural UniversityNanjing, China; ^3^Department of Biology, University of North CarolinaCharlotte, NC, USA

**Keywords:** genetic relationship, phosphorus efficiency, photosynthetic traits, QTL analysis, high-density genetic map, soybean

## Abstract

Plant productivity relies on photosynthesis, and the photosynthetic process relies on phosphorus (P). The genetic basis of photosynthesis and P efficiency (PE) affecting yield has been separately characterized in various crop plants. However, the genetic relationship between PE and photosynthesis remains to be elucidated. In this study, we used a combined analysis of phenotypic correlation, linkage mapping, and expression analysis to dissect the relationship between PE and photosynthesis. We found significant phenotypic correlations between PE and photosynthetic related traits, particularly under low P stress. A total of 172 QTLs for both traits were detected and classified into 29 genomic regions. 12 (41.4%) of 29 regions were detected to be associated with both PE and photosynthetic related traits. Three major QTLs, *q14-2, q15-2*, and *q19-2*, were found to be associated with both traits and explained 6.6–58.9% of phenotypic variation. A photosynthetic-specific QTL cluster, *q12-1*, was detected under both normal and low P conditions, suggesting that genes responsible for this region were less effected by low P stress, and could be used in high photosynthetic efficiency breeding programs. In addition, several candidate genes with significantly differential expression upon low P stress, such as a purple acid phosphatase gene (*Glyma.19G193900*) within *q19-2* region, were considered as promising candidates involved in regulating both soybean PE and photosynthetic capacity. Our results reveal a significant genetic relationship between PE and photosynthetic traits, and uncover several major genomic regions specific or common to these traits. The markers linked closely to these major QTLs may be used for selection of soybean varieties with improved P efficiency and photosynthetic capacity.

## Introduction

Phosphorus (P) is essential for all living cells and organisms. Low P stress represents a major constraint on plant growth and yield worldwide (Zhang et al., 2014). In past decades, P fertilizers have enormously increased crop yields. However, greater than 50 million tons of consumable P is estimated to be used for global agricultural practices annually and causes serious effects to the environment, including soil acidification, and water pollution (Van Vuuren et al., 2010). Moreover, global commercial phosphate reserves may be depleted within a few decades and there is currently no known substitute (Cordell and White, 2011). The production and conservation of P create an enormous challenge for sustaining global food production in the foreseeable future. To response to “The Phosphorus Crisis” (Vaccari, 2009) and simultaneously ensure food security and environmental quality, identification and dissection of P-efficient genes represent the important steps for the subsequent development of P-efficient crops, securing sustainable food supply and agriculture (Gaxiola et al., 2012).

Soybean is an important crop providing the primary source of plant protein and vegetable oil for human and livestock consumption. Soybean is also a high P demand species but sensitive to low P stress. Low P stress has been the largest constraint affecting soybean production compared to other nutrient deficiencies, toxicities, or diseases (Gowin, 1997). In recent years, in order to understand the genetic basis of soybean P efficiency, great efforts have been made in evaluating P-efficient soybean genotypes, and applying linkage/association analyses to map genes underlying or markers linked to soybean P efficiency. Significant genetic variation in the soybean germplasms suggests that the selection of better P-efficient varieties can be achieved by breeding processes (Li et al., 2005; Zhang et al., 2009, 2014; Liang et al., 2010). In our recent study, a key QTL gene, *GmACP1* encoding an acid phosphatase, underlying P efficiency was identified (Zhang et al., 2014). Overexpression of *GmACP1* in soybean plants significantly increased P efficiency by 11–20% compared with controls. However, the yield of these transgenic plants was not significantly increased, which was mainly attributed to the photosynthetic capacity is not prominent, while photosynthesis is the basis of crop growth and yield formation. Thus, it is very important to evaluate soybean photosynthetic capacity when in cultivating P-efficient soybean varieties.

Crop yield relies on photosynthesis, and the photosynthetic process is largely relies on P containing compounds (Veneklaas et al., 2012). P plays a very important role in the composition of chloroplast and photosynthesis and can be directly involved in the assimilation of photosynthesis and photosynthetic phosphorylation (Balemi and Negisho, 2012). Thus, photosynthesis is sensitive to low P stress. Generally, most common reports state that P deficiency reduces photosynthetic capacity through: (i) directly affecting ATP production (Rao and Terry, 1995), (ii) inactivation of enzymes involved in the ribulose bisphosphate (RuBP) regeneration (Fredeen et al., 1990), (iii) inactivating RuBP carboxylase enzyme which catalyzes CO_2_ fixation, (iv) combined effect of ii and iii (Brooks et al., 1988). For example, studies have reported that P status in plant tissue directly influences photosynthetic metabolic processes of energy transfer (ATP, NADPH), regeneration of substrates and utilization of photosynthates and CO_2_ diffusion inside the leaves (Brooks et al., 1988; Singh et al., 2013). Due to the down regulation of the photosynthesis under P deficiency, plants may be starved for carbohydrates, leading to smaller plant stature and leaf area, thus reduced growth and yield (Høghjensen et al., 2002).

In our recent experiments, however, we have found that some soybean germplasms could maintain a high photosynthetic capacity even under low P stress. Several previously studies have also reported that high P efficiency (high yield or relative growth rate per unit of P) of a plant species/genotypes under P deficiency can be related to higher net carbon fixation (Li et al., 2006) achieved possibly through effective cytoplasmic P homeostasis or through selectively allocating more P to the cytoplasm. Higher P efficiency under P limiting condition may also be caused due to lower carbon demand for root respiration (Nielsen et al., 2001). In soybean, however, it is less clear that how low P concentration in P-limited systems affects photosynthetic biochemistry. Therefore, evaluating the responses of plant physiological processes to low P stress is important to understand the underlying mechanisms of photosynthetic limitations.

Both P efficiency (PE) and photosynthetic related traits are complex traits, depending on both genetic and environmental factors and their interactions. In soybean, poor understanding of the genetic basis of P efficiency as well as limited knowledge about photosynthetic related traits and its relationship with PE have hindered the selection of varieties with high PE and high photosynthetic efficiency. Mapping quantitative trait loci (QTLs) has been a powerful tool to identify genomic regions or candidate genes involved in genetic variation of complex traits. Many QTLs underlying soybean PE related traits have been identified, such as biomass traits (Li et al., 2005), root architecture (Ao et al., 2010), P concentration, acid phosphatase activity (Zhang et al., 2014), and flower/pod abscission rates (Zhang et al., 2010). Likewise, several soybean QTLs that regulate photosynthetic related traits have been identified (Yin et al., 2010a,b; Hao et al., 2012). Despite these extensive advancements in the genetic knowledge of PE and photosynthetic related traits, it is unclear if genetic relationships between, and common QTLs for, both traits exist. Given that better understanding the genetic relationship between PE and photosynthesis traits, and efficient use of P in photosynthesis is a potentially important factor for sustainable crop production. Therefore, a deep investigation of photosynthesis traits under low P stress is required to directly uncover the genetic relationship between PE and photosynthetic related traits.

In the present study, a soybean RIL population derived from a cross of two parental lines Nannong94-156 and Bogao with contrasting PE and photosynthetic traits was evaluated. Phenotyping of PE and photosynthetic related traits in response to two P levels was performed for seedling plants in hydroponics and adult plants in pot trials, respectively. The objectives of this study were (a) investigate the phenotypic association between PE and photosynthetic traits under normal P (NP) and low P (LP) conditions, (b) determine the genetic basis of the relationship between both traits through QTL analyses, (c) identify molecular markers associated with photosynthetic specific-QTL not affected by low P stress, and the markers linked to overlapped QTLs for the both traits, which may be helpful in improving both PE and photosynthesis at a time, and (d) prediction of possible candidate genes for further study.

## Materials and methods

### Plant materials

A segregating soybean population consisting of 152 RILs derived from a cross between varieties “Nannong94-156” (male parent) that possessed high P efficiency and “Bogao” (female parent) with low P efficiency, was used to map QTLs for PE and photosynthetic traits. In our previous studies, Nannong94-156 was characterized as a P-efficient variety and Bogao was a P-inefficient variety (Zhang et al., 2009). In addition, two parent accessions exhibited huge variation in photosynthetic related traits (Cui and Yu, 2007; Hao et al., 2012).

### Hydroponics experiments

Two parental lines and 152 RILs were hydroponically grown and evaluated by two independent experiments in Henan Agricultural University in 2013 and 2014 (Hydroponic experiments, E1 and E2) under controlled conditions in artificial climate chamber (28/20°C day/night temperature, 10 h light/14 h dark photoperiod). The hydroponic tank is 70 × 50 × 30 cm (LWH) containing 60 holes, with two plants each hole. Approximately 100 l of nutrient solution installed in a tank, allowing 0.8 l of hydroponic solution per plant. Preparation of seeds for germination and treatment was conducted as previous described (Zhang et al., 2014). Briefly, the seeds were surface-sterilized with chlorine and germinated in sterile vermiculite. The uniform soybean seedlings with fully-expanded cotyledons were selected and transferred into modified one-half Hoagland's nutrient solution supplemented with 500 μM P (normal P, NP, KH_2_PO_4_) for 3 days. Half of the seedlings were then transferred to modified one-half Hoagland's nutrient solution lacking P (5 μM P, low P, LP) for 14 days, while the other half remained in normal P condition as controls (pH = 5.8 and the solution was renewed every 3 days). The soybean plants were placed in the hydroponics box using a completely randomized block design. The value of each replicate was represented by the means of three seedlings for each RIL or six seedlings for each parental line. Roots were separated from the shoot and stored at −20°C freezer before the measurements as described in Table 1. The roots and shoots were deactivated by heating-induced denaturation at 105°C for 60 min; and then oven-dried at 70°C until a constant weight, and root dry weight (RDW), shoot dry weight (SDW), and root to shoot ratio (R/S) were evaluated. The dried samples were milled and subsequently digested with concentrated H_2_SO_4_ and H_2_O_2_ to facilitate the determination of P concentration (PC) using the molybdate-blue colorimetric method (Murphy and Riley, 1962). P use efficiency in the plant (PUE) was defined as the mg of plant dry weight produced per mg of P absorbed by plants, while P uptake (Pup) was defined as total P in the plant (mg plant^−1^; Table 1).

**Table 1:** **Summary of the investigated traits in this study and the measurements**.

**Classification**	**Trait**	**Abb.^a^**	**Units**	**Trait measurements**	**Measured environments**
PE-related traits	Root dry weight	RDW	g Plant^−1^	Dried and weighted using a balance (1/1000 g)	H
	Shoot dry weight	SDW	g Plant^−1^	Dried and weighted using a balance (1/1000 g)	H
	Total dry weight	TDW	g Plant^−1^	RDW+SDW	H
	Root to shoot ratio	R/S	mg/mg	Root dry weight/shoot dry weight	H
	Phosphorus concentration	PC	mg g^−1^	Olsen method	H/F^b^
	Phosphorus uptake	Pup	g Plant^−1^	TDW × PC	H/F
	Phosphorus use efficiency	PUE	mg g^−1^	1/PC	H/F
	Biomass yield	BY	g Plant^−1^	Dried and weighted using a balance (1/1000 g)	F
Photosynthetic related traits	Chlorophyll content	CC	μg ml^−1^	Average of the five plants (SPAD value)	H/F
	Net photosynthetic rate	Pn	μmol·m^2^·s^−1^	Average of the three plants (LI-6400)	H
	Transpiration rate	Tr	g·m2·h^−1^	Average of the three plants (LI-6400)	H
	Stomatal conductance	Co	mmol·m^−2^· s^−1^	Average of the three plants (LI-6400)	H
	Intercellular carbon dioxide concentration	Ci	μL·L^−1^	Average of the three plants (LI-6400)	H

a Abbreviations.

b H denotes the given trait has been measured in hydroponics and F denotes in field.

Photosynthetic related traits including net photosynthetic rate (PN), transpiration rate (Tr) and intercellular carbon dioxide concentration (Ci) and stomatal conductance (Co) were measured using a LI-6400 portable photosynthesis system (Li-Cor Inc., Lincoln, NE, USA). Chlorophyll content (CC) was measured with a chlorophyll meter (CCM-200, OptiSciences, Inc., USA). The upper third leaf of three plants per plant from three replications at the V2 development stage (Second trifoliolate stage) was used for phenotyping. Three plants from two replications per genotype were determined.

### Field experiments

Three greenhouse trials for 152 RILs and two parent lines were performed at different environments, including the Nanjing Agricultural University (Nanjing, China) experimental station in Jiangpu (32.1°N 118.4°E) in 2008 (designated as E3), Henan Agricultural University experimental station in Maozhuang (34.8°N 113.6°E) in 2008 and 2011 (designated as E4 and E5). The experiment was conducted in a randomized block design with a split-plot arrangement. The main plots were treatment (normal P and low P); subplots were 154 genotypes (2 parents plus 152 lines). There were two replicates with six plants in each replicate. Seeds of each genotype were sown in a 10 L pot filled with 4 kg dry soil, with two plants per pot. The soil had a very low content of phosphorus, with average 5.22 mg kg^−1^ available P, 0.28 g kg^−1^ of total nitrogen, 46.8 mg kg^−1^ of available K and 12.5 g kg^−1^ of organic matter. To evaluate plant responses to low P availability, solution containing 60 mg kg^−1^ H_2_NCONH_2_ and 20 mg kg^−1^ KCl (20 mg kg^−1^) was applied to low P treatment pots. For the normal P treatment, solution containing 60 mg kg^−1^ H_2_NCONH_2_ and 20 mg kg^−1^ KH_2_PO_4_ were applied. All pots were regularly watered as needed. Fertilizer was applied four times during the experiment, prior to planting and at the seedling, flowering and seed filling stages. At R6 stage (full seed stage), the CC, PC, Pup, and PUE of healthy plants within each row (3–5 individuals) were determined for the measurements as described in Table 1. Plant tissues above ground were oven-dried in determining biomass yield (BY).

### Data analysis and candidate genes prediction

Phenotypic data was analyses with software SAS 9.0 (SAS Institute Inc., NC, USA) using the GLM procedure. Combinations of year-location were treated as environments (E). Genotype (G) was treated as fixed, and E and interaction of genotype-by-environment (G × E) as random. The procedure LSMEANS was then performed to estimate phenotype values for the genotypes that were used for the subsequent phenotypic analysis and correlation analysis. The procedure VARCOMP was conducted to estimate genotypic variance (σ^2^G) (σG^2^), G and E interaction variance (σ^2^G × E) (σG × E^2^) and error variance (σ^2^e) (σe^2^). Pearson correlation coefficients and PCA were calculated using SPSS Statistics 17.0 (SPSS, Inc., Chicago, IL, USA) and further visualized using the R package (Venables and Smith, 2005).

The linkage map used in this study was constructed as previously described (Zhang et al., 2016). This map, spanning 3020.59 cM in length, contained 6159 SNP markers on 20 chromosomes, with an average distance of 0.49 cM between adjacent markers. The additive and epistatic QTLs underlying the PE and photosynthetic related traits at different P levels were identified by the QTL IciMapping program v4.0 using single environment phenotypic values (Wang et al., 2014). Briefly, for the additive QTL, the inclusive composite interval mapping (ICIM) method was used in the software, the *P*-values for entering variables (PIN) and removing variables (POUT) were set at 0.01 and 0.02, and the scanning step was 2 cM. The ICIM-EPI method was used to detect epistatic QTL, the PIN and POUT were set at 0.0001 and 0.0002, respectively, and the scanning step was 5 cM. The LOD thresholds for each index of QTL were determined by 1000 permutation test at 95% confidence level. The proportion of observed phenotypic variance explained by each additive QTL or epistatic QTL and the corresponding additive effects were also estimated.

The predicted genes in the target QTL regions were analyzed according to the annotation of the soybean reference genome (Wm82.a2.v1) in Phytozome v.11 (https://phytozome.jgi.doe.gov). Functional predictions of genes were manually confirmed by blastp function in NCBI (http://www.ebi.ac.uk/Tools/sss/ncbiblast/). In addition, GO enrichment analysis of predicted genes was performed using the GO online analyses with default setting (http://bioinfo.cau.educn/agriGO/analysis.php; Du et al., 2010).

### qRT-PCR analysis

Leaf tissues of four representative soybean accessions (NN94-156, Bogao, Suxie1, and Kefeng1) were sampled 7 days post low P stress treatment and stored at −70°C freezer until use. The total RNA was isolated from the leaves using the RNA simple Total RNA Kit (DP419, TIANGEN, Beijing, China) and treated with 10 units of RNase-free DNase I (TaKaRa, Japan). The first strand of cDNA was synthesized using the SuperScript III First Strand Synthesis System (Invitrogen, USA). Gene expression was determined by qRT-PCR assays using the ABI 7500 system (Applied Biosystems, Foster City, USA). The PCR reactions contained 50 ng of the first cDNA strand, 0.5 μL of 10 μmol L^−1^ gene-specific primers (5′-GCGTGCGTGTACAAATTGTGA/ATTGTGTTATCTTGCAGC AA CGA-3′) for *Glyma.19G193900*, and 10 μL of the real-time PCR SYBR MIX (QPK-201; TOYOBO). The PCR conditions were as follows: 95°C for 5 min and 40 cycles at 95°C for 15 s and 60°C for 60 s. The soybean tubulin gene (GenBank: AY907703.1) was amplified as a control, and a negative control reaction was performed using water instead of the cDNA. Three technical replicates were performed for each reaction, and the data were analyzed using the ABI 7500 Sequence Detection System (SDS) software version 1.4.0. The normalized expression, reported as fold changes, was calculated for each sample as ^ΔΔ^CT = (^CT^, Target^−CT^, Tubulin) genotype-(^CT^, Target^−CT^, Tubulin) calibrator (Livak and Schmittgen, 2001).

## Results

### Significant variation in PE and photosynthetic related traits in the soybean RILs

**Figure 1:**
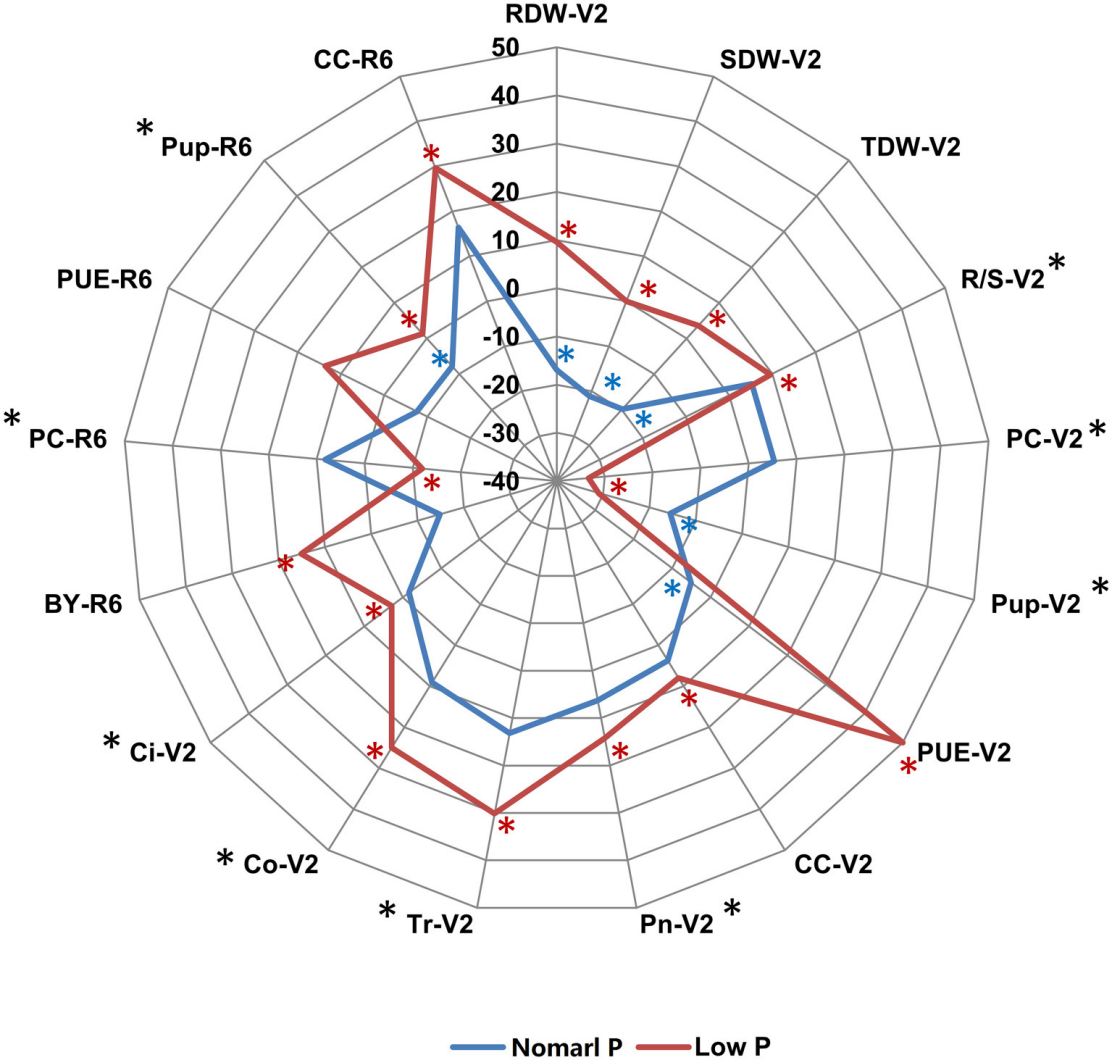
**The phenotypic difference between the two parental lines Nannong94-156 and Bogao under NP and LP conditions**. The difference for each trait is indicated by the relative increase or decrease of Nannong94-156 to Bogao, which was calculated as (Nannong94-156–Bogao)/Bogao × 100%. The blue and red solid lines represent the percentages under NP and LP levels, respectively. A significant difference between the two parents at NP level is labeled by a blue asterisk and at LP level by a red asterisk. Traits that showed a significant difference between two P levels are labeled by a black asterisk (*P < 0.05, *t*-test).

To determine the genetic variation of PE and photosynthetic traits in soybean plants, we evaluated these phenotypic traits in two independent hydroponic experiments and three pot trials in field under both normal P (NP) and low P (LP) growth conditions. In hydroponic experiments, seven PE related traits (RDW, SDW, TDW, R/S, PC, Pup, and PUE) and five photosynthetic related traits (CC, Pn, Tr, Co, and Ci) during soybean seedling stage (V2) were determined using 152 soybean RILs. Under both NP and LP conditions, the parental line Bogao showed higher SDW, TDW, and Pup than those in parental line Nannong94-156 (Figure 1, Table 2), which was consistent with the fact that Bogao was a high biological-yielding soybean variety (Cui et al., 2008). In contrast, SDW in Nannong94-156 reduced 12.9% due to LP stress, but by more than double that (31.4%) in Bogao, suggesting that Nannong94-156 might be more tolerant to low P stress (Figure 1, Table 2). Accordingly, a significant decrease of 71.3 and 76.6% of total P accumulation in Nannong94-156 and Bogao were, respectively, observed because P concentration in both varieties was decreased by up to 61.4 and 75.5%, respectively. Importantly, Nannong94-156 had approximately twice times higher PUE than that in Bogao under low P conditions, indicating that Nannong94-156 was a LP stress tolerant variety (Figure 1, Table S1). In addition, R/S in Nannong94-156 was higher than in Bogao under both P levels and trials, which may enhance the ability of root absorption of P, leading to the ground part of the plants less affected by low P stress. Under LP conditions, TDW in both parental lines decreased by 25.8 and 4.2%, respectively.

**Table 2:** **Descriptive statistical results for PE and photosynthetic related traits in soybean recombinant inbred lines (RILs) and their parents across experiments conducted under NP and LP conditions**.

**TraitName^a^**	**Normal P**						**Low P**						**G^c^**	**T^d^**	**R^e^**	G*E^f^
	**Parents**		**RILs**				**Parents**		**RILs**							
	**Bogao**	**94156**	**Mean**	**Range**	**CV%**	***h*^2^%^b^**	**Bogao**	**94156**	**Mean**	**Range**	**CV%**	***h*^2^%**				
SDW-V2	1.6	1.3	3.9	2.58–5.43	13.9	74.1	1.4	1.5	2.8	1.83–4.1	15.3	63.2	^**^	^**^	NS	^**^
RDW-V2	3.2	2.5	1.6	1.27–1.95	8.3	69.9	2.2	2.2	1.6	1.23–2.09	10.3	67.2	^**^	^**^	NS	^**^
TDW-V2	4.8	3.9	5.3	3.78–7.19	12.3	67.6	3.6	3.7	4.3	3.04–5.9	12.1	63.6	^**^	^**^	NS	^**^
RS-V2	0.5	0.5	0.4	0.32–0.53	10.1	73.5	0.6	0.7	0.6	0.42–0.88	14.8	67.8	^**^	^**^	NS	^**^
PC-V2	1.3	1.4	1.5	0.19–3.29	37.6	71.5	0.5	0.3	0.5	0.05–1.55	66.6	66.5	^**^	^**^	NS	^**^
Pup-V2	6.3	5.4	7.5	0.96–16.5	38.5	58.8	1.8	1.3	1.9	0.22–6.85	64.7	59.2	^**^	^**^	NS	^**^
PUE-V2	0.8	0.7	0.9	0.34–11.4	65.5	50.6	2.0	2.9	3.4	0.65–20.5	65.7	44.6	^**^	^**^	NS	^**^
CC-V2	13.3	13.8	12.3	8.49–17.9	12.2	65.8	11.2	12.1	12.0	9.6–16.78	9.9	62.1	^**^	^**^	NS	^**^
Pn-V2	18.2	19.4	21.4	12.65–34.1	19.2	67.7	15.4	17.6	17.1	10.7–23.9	18.5	61.3	^**^	^**^	NS	^**^
Co-V2	4.3	4.9	0.4	0.06–0.8	36.1	63.7	3.1	4.1	0.2	0.08–0.32	33.9	55.6	^**^	^**^	NS	^**^
Tr-V2	0.4	0.5	4.3	2.7–6.0	18.0	61.1	0.2	0.2	3.5	1.65–6.08	26.2	56.9	^**^	^**^	NS	^**^
Ci-V2	733.0	721.0	745.7	589–871.3	9.6	66.0	756.0	778.0	755.8	579.2–881	10.0	65.2	^**^	^**^	NS	^**^
CC-R6	43.2	36.8	23.8	17.2–32.8	14.3	40.0	27.1	31.2	21.5	14.3–29.2	13.7	44.3	^**^	^**^	NS	^**^
PC-R6	1.8	2.0	2.0	0.8–3.6	24.5	65.2	1.3	1.1	0.9	0.46–1.78	27.3	63.2	^**^	^**^	NS	^**^
BY-R6	0.5	0.5	43.6	26.8–68.9	16.8	55.3	0.8	0.9	36.4	21.8–55.8	16.0	57.2	^**^	^**^	NS	^**^
Pup-R6	78.6	72.5	85.1	29.1–170.9	32.2	61.2	33.9	34.3	32.5	13.9–69.1	33.5	59.8	^**^	^**^	NS	^**^
PUE-R6	17.4	20.2	0.6	0.3–1.3	28.9	52.3	15.0	19.5	1.2	0.63–2.37	25.4	55.6	^**^	^**^	NS	^**^

a TraitName is a composite of the influenced trait: root dry weight (RDW), shoot dry weight (SDW), total dry weight (TDW), root to shoot ratio (R/S), P concentration (PC), phosphorus uptake (Pup), phosphorus use efficiency (PUE), biomass yield (BY), net photosynthetic rate (PN), transpiration rate (Tr) and intercellular carbon dioxide concentration (Ci), stomatal conductance (Co) and chlorophyll content (CC) followed by the treatments, environments, and growth stages (V2, second trifoliolate stage; R6, full seed stage). See Table 1 for an explanation of trait abbreviations and their units.

b h^2^ (%), broad-sense heritability;

c genotype;

d treatment;

e replication;

f genotype × environments. Significant difference is indicated by an asterisk (

** P < 0.01). NS: not significant.

In pot experiments in the field, four PE related traits (BY, PC, Pup, and PUE) and CC during soybean R6 stage was determined in 152 soybean RILs across three environments. In general, phenotypic changes were not as much in the pot experiments under low P condition compared with hydroponic experiments. BY in Bogao was higher than in Nannong94-156 at both P levels. Similar to the hydroponic experiment, PUE in Nannong94-156 was higher than that in Bogao under low P conditions (Figure 1, Table 2). Pup of both lines decreased by 56.9 and 52.7%, but the differences were not significant. However, a variation of CC in response to LP stress in both parental lines was also observed, with a significantly decrease of 13.6% in Bogao, and only 3.8% in Nannong94-156.

Within the RIL population generated from a cross between Nannong94-156 and Bogao, we found that the PE and photosynthetic traits were extensively segregated and normally distributed, showing a considerable phenotypic variation with a wide range of coefficient of variation (CV) ranging from 8.3 to 66.6% (Table 2, Table S1). As shown in Table 2, Table S1, average TDW and BY was significantly affected by LP stress, with decreases of 18.9, 16.5, 74.6 in TDW, BY, and Pup, respectively. However, average R/S and PUE showed significantly increased, with 46.3% for R/S, 121.8 and 274.7% for PUE in field and hydroponics, respectively. Accordingly, photosynthetic related traits, Pn, Tr, and Co, were decreased by up to 20.3, 17.1, and 55.0%, respectively, across environments. For each measured trait, significant effects were observed for genotype, P levels, environments, and their corresponding interactions, indicating strong G × E interaction (Table 2). Nevertheless, the heritability (*h*^2^) of PE related traits were rather high, varying from 50.6 to 74.1% under NP and from 44.6 to 67.8% under LP (Table 2). The heritability (*h*^2^) of photosynthetic related traits was moderate, ranging from 40.0 to 67.7% under NP and from 44.3 to 63.2% under LP condition (Table 2).

### Phenotypic relationship between PE and photosynthetic related traits

**Figure 2:**
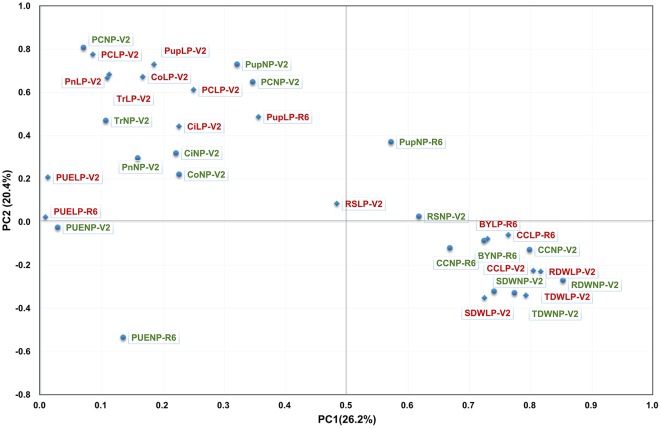
**PCA of the RIL population for PE related traits (in red) and for photosynthetic related traits (in green) under the conditions of NP (circle) and LP (diamond) levels**. Thirty-four traits were projected onto the first and second principal components.

To investigate the contribution of P to plant photosynthesis, the Pearson correlation between PE and photosynthetic related traits was determined, and a PCA was performed to visualize the correlation (Figure 2, Table S2). Overall, correlations between PE and photosynthetic related traits were much lower than those within PE or photosynthetic related traits. However, significant correlations were observed between photosynthetic traits, such as CC, Pn, Tr with PC, Pup, PUE, TDW, and BY. Moreover, the correlation coefficient was higher under LP than in NP condition (Figure 2, Table S2). PCA demonstrated that all of photosynthetic traits except CC were most closely related with PC and Nup under both NP and LP conditions (Figure 2), indicating that PC and Pup is more likely to be genetically associated with photosynthetic traits. Irrespective of P levels, Pn, Tr, and Co appeared to be more related to PC and Pup than those biomasses related traits (Table S2), and the correlation coefficient significantly greater under LP conditions. In addition, these results also suggest that some photosynthetic related traits have a closer relationship with PC and Pup than they do with PUE, and this relationship was even strengthened under LP condition.

### Detection of QTLs for PE and photosynthetic related traits

**Figure 3:**
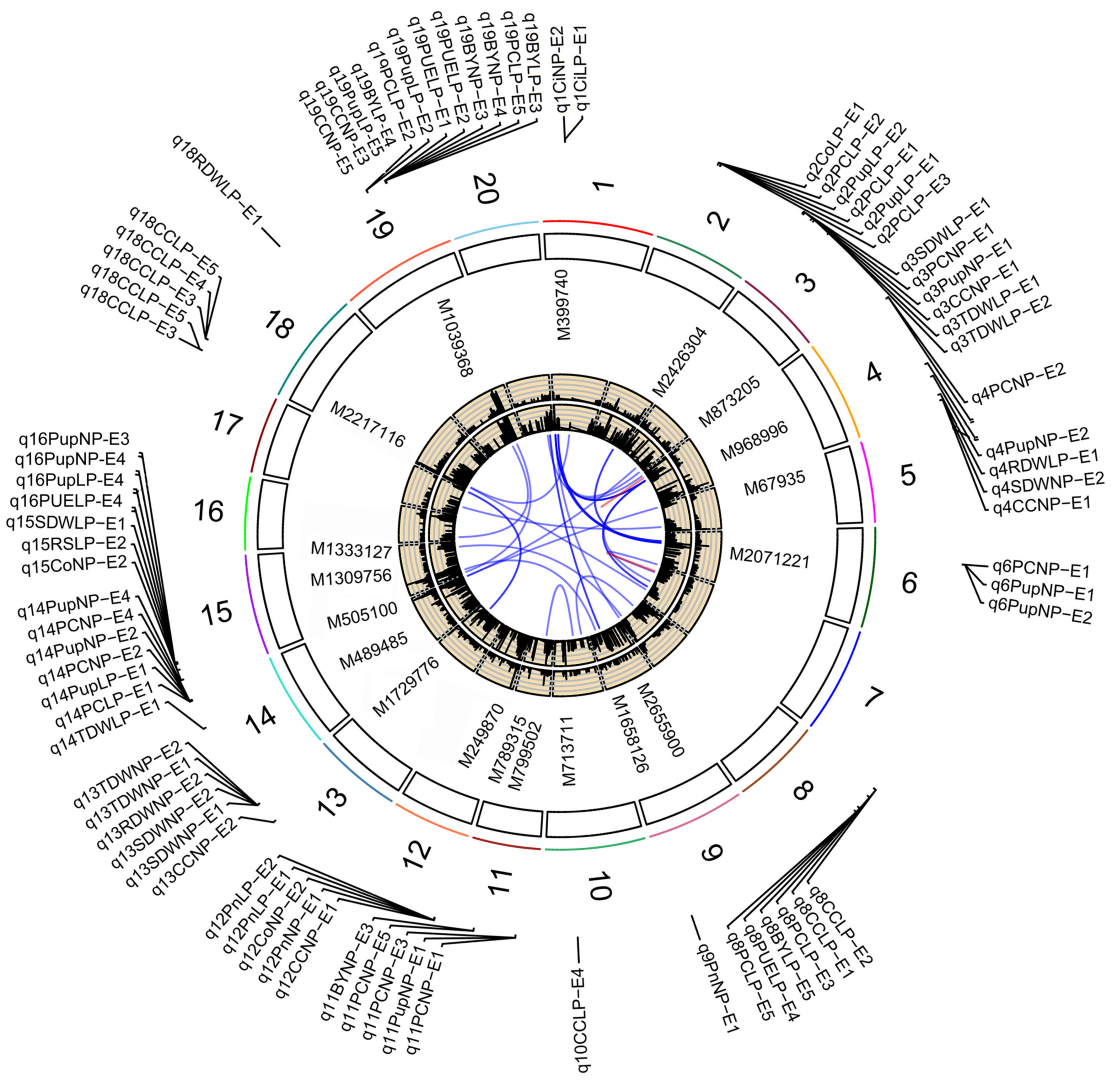
**Soybean chromosomes and main QTLs for investigated traits under Normal P (NP) and low P (LP) conditions**. The lines link denotes epistatic associations between QTL and QTL. Blue line denotes two QTLs in different chromosomes, while red line denotes two QTLs in the same chromosome. The outside/inside wheat-colored circle indicates the LOD/PVE value curve for investigated traits across environments. The outermost circle indicates the 20 soybean chromosomes, main QTLs for investigated traits under normal P (NP) and low P (LP) conditions and the position and linked markers of these QTLs on the chromosomes.

Based on the high-density genetic map, a total of 172 QTLs across all 20 soybean chromosomes, except the 7, 17, and 20 chromosomes, for all investigated traits through all independent experiments were identified in the RIL population (Figure 3, Figure S1, Table S3). Among 172 QTLs, 98 QTLs for seven PE related traits and five photosynthesis related traits were detected in hydroponics and 74 QTLs for four PE related traits and chlorophyll content were detected in field experiments (Figure 3, Figure S1, Table S2). More QTLs (91) were detected associated with both PE and photosynthesis related traits under LP conditions, while 81 QTLs were detected under NP conditions (Figure 3, Figure S2). Within the identified QTLs for photosynthetic traits, a similar proportion of QTLs carried the favorable alleles that were originated from either the parental line Nannong94-156 or Bogao. By contrast, ~80% of identified QTLs associated with PE related traits had the favorable alleles from the low P tolerant parent Nannong94-156. Total phenotypic variation explained by the QTLs for each PE related trait ranged from 5.3 to 25.1%, and those for each photosynthetic related trait ranged from 5.4 to 72.9% (Table S3).

In this study, overlapped or adjacent QTLs with less than 5 Mb were classified into the same loci (Visscher et al., 1996; Öckinger et al., 2006; Swanson-Wagner et al., 2009; Wang et al., 2012). Base on this rule, 172 QTLs were classified into 29 genomic regions (loci; Table 3). Among the 29 loci, most loci were identified under both NP and LP conditions, such as those QTLs on chromosomes 2, 3, 4, 12, 14, 15, and 19. For example, *q14-2* was detected to be associated with PE and photosynthetic related traits across environments, including PC, Pup, PUE, biomass, Ci, and Pn. The LOD scores of the QTLs at this locus ranged from 2.57 to 9.29, with average LOD of 4.59, and these QTLs could explain 6.83–33.74% of phenotypic variance (Table S3). These QTLs most likely contributed significantly to the genetic basis of PE and photosynthetic related traits, and were relatively less influenced by environmental stress. On the other hand, some QTLs that were detected uniquely to either NP or LP conditions were represented as NP-specific or LP-specific QTLs, such as QTLs, *q4-1, q4-2, q6, q9*, and *q13*, on chromosomes 4, 6, 9, and 13 were detected only under NP conditions. By contrast, several LP-specific QTLs, *q2-1, q5, q8-1, q8-2*, and *q18-1*, on chromosomes 2, 5, 8, and 18 were detected only under LP conditions, which suggested that the genes underlying these QTLs could be more favorably induced by low P stress. Further dissection of these P condition-specific-QTLs may increase our understanding of the genetic basis of PE and photosynthetic related traits.

**Table 3:** **The characters of 29 consensus loci associated with PE and photosynthetic related traits across environments and treatments**.

**Name^a^**	**Traits-years-treatments^b^**	**Chr.^c^**	**Marker interval^d^**	**Position^e^**	**LOD^f^**	**PVE(%)^g^**
*q1*	CiLP-E1, CiNP-E2	1	Marker399740–Marker405817	984367–984667	2.86	12.28
***q2-1***	**CoLP-E1, PCLP-E2, PupLP-E2, PCLP-E1, PupLP-E1, PCLP-E3**	**2**	**Marker2426304–Marker2355122**	**13942698–15102489**	**3.76**	**14.04**
*q2-2*	RSNP-E2, TDWNP-E2, CCNP-E1, CCLP-E5	2	Marker2359119–Marker2407344	41077137–45184430	4.02	9.16
***q3-1***	**SDWLP-E1, PCNP-E1, PupNP-E1, CCNP-E1, TDWLP-E1**	**3**	**Marker873205–Marker923093**	**2384930–7764196**	**3.19**	**8.39**
*q3-2*	TDWLP-E2, CCNP-E2, CCLP-E1, CCLP-E2	3	Marker968996–Marker945189	15406533–18840627	3.70	8.68
*q3-3*	CCNP-E1	3	Marker953521–Marker863963	38757448–38757723	3.09	5.56
*q4-1*	PCNP-E2, PupNP-E2	4	Marker70489–Marker94279	2244572–2244846	3.52	11.16
*q4-2*	PCNP-E4, PupNP-E2	4	Marker67935–Marker129026	32704336–38198190	3.16	8.62
***q4-3***	**RDWLP-E1, SDWNP-E2, SDWLP-E1, SDWNP-E1, RDWNP-E1, RDWNP-E2, TDWNP-E1, TDWNP-E2, SDWLP-E2, TDWLP-E2, CCNP-E1**	**4**	**Marker101824–Marker58600**	**46024793–50216432**	**4.44**	**10.86**
*q5*	CCLP-E3, SDWLP-E2	5	Marker1911126–Marker1840642	5023300–5023579	4.30	9.16
*q6*	PCNP-E1, PupNP-E1, PupNP-E2	6	Marker2071221–Marker2174814	15623936–15624197	4.02	11.22
*q8-1*	CCLP-E1, CCLP-E2, PCLP-E3	8	Marker2655981–Marker2726667	1796491–5706738	4.12	8.89
***q8-2***	**BYLP-E5, PUELP-E4, PCLP-E5, PupLP-E5, PUELP-E5**	**8**	**Marker2655900–Marker2651744**	**11520732–13520574**	**3.55**	**8.01**
*q9*	PnNP-E1	9	Marker1658126–Marker1641616	43233332–43233607	3.54	9.82
*q10*	CCLP–E4	10	Marker713711–Marker700268	36602559–36602814	3.31	9.50
*q11-1*	PCNP-E1, PupNP-E1, PCNP-E3	11	Marker799502–Marker844658	7615924–7616190	5.33	15.96
***q11-2***	**PCNP-E5, BYNP-E3, BYNP-E4, BYNP-E5, PupNP-E5, PUENP-E5, BYLP-E3, BYLP-E5, PupLP-E5**	**11**	**Marker789315–Marker822756**	**24450424–24450687**	**3.22**	**7.18**
***q12-1***	**CCNP-E1, PnNP-E1, CoNP-E2, PnLP-E1, PnLP-E2, CoLP-E2, CCNP-E2, CCLP-E1, CCLP-E2**	**12**	**Marker249870–Marker262276**	**3008087–4610598**	**7.37**	**23.74**
*q12-2*	PnNP-E2, CCLP-E3	12	Marker200457–Marker203972	31303690–31303972	3.05	13.78
***q13***	**CCNP-E2, SDWNP-E1, SDWNP-E2, RDWNP-E2, TDWNP-E1, TDWNP-E2**	**13**	**Marker1729776–Marker1778051**	**35339389–40660749**	**4.22**	**10.41**
*q14-1*	TDWLP-E1	14	Marker489485–Marker557885	33522076–33522349	3.83	9.26
***q14-2***	**PCLP-E1, PupLP-E1, PCNP-E2, PupNP-E2, PCNP-E4, PupNP-E4, PUENP-E4, PupNP-E1, PCNP-E1, CiNP-E2, PCNP-E3, CiLP-E1, PCLP-E4, PupLP-E4, PUELP-E4, PupLP-E5, PnLP-E2, PCLP-E5, PUELP-E5, PCNP-E5, BYNP-E3, BYNP-E4, BYNP-E5, PupNP-E5, PUENP-E5, BYLP-E3, BYLP-E4, BYLP-E5**	**14**	**Marker505100–Marker509340**	**45573911–47652816**	**4.59**	**14.66**
*q15-1*	CoNP-E2, RSLP-E2	15	Marker1309756–Marker1323793	2943851–7164361	3.54	10.18
***q15-2***	**SDWLP-E1, SDWNP-E2, RDWNP-E2, TDWLP-E1, TDWNP-E1, TDWNP-E2, CoLP-E1, SDWNP-E1, RDWNP-E1, TrNP-E1, TrNP-E2, CiNP-E1, SDWLP-E2, TDWLP-2, PUELP-E2, PCLP-E3, PupLP-E3, PUELP-E3**	**15**	**Marker1333127–Marker1381067**	**9586975–11346054**	**6.04**	**20.00**
*q16*	PupNP-E3, PupNP-E4, PupLP-E4, PUELP-E4	16	Marker1146835–Marker1164294	31778047–31778323	3.01	12.21
***q18-1***	**CCLP-E5, CCLP-E3, CCLP-E3, CCLP-E4, CCLP-E5**	**18**	**Marker2217116–Marker2237156**	**2037678–2889549**	**8.36**	**22.00**
*q18-2*	RDWLP-E1	18	Marker2270259–Marker2325242	53977091–53977334	4.16	12.73
*q19-1*	PCLP-E2, PupLP-E2	19	Marker1039368–Marker1043830	36624688–36624978	3.43	11.47
***q19-2***	**PUELP-E1, PUELP-E2, BYNP-E3, BYNP-E4, PCLP-E5, BYLP-E3, BYLP-E4, PupLP-E5, CCNP-E3, CCNP-E4, CCNP-E5, PCNP-E5, BYNP-E5, PupNP-E5, BYLP-E5, PUELP-E5, PCLP-E1, PCLP-E2, PupLP-E1, PupLP-E2, PCLP-E3, PCLP-E4, PUELP-E3, PUELP-E4, CiLP-E2, PUENP-E5, TrLP-E1, TrLP-E2**	**19**	**Marker1094659–Marker1066605**	**44865752–49307740**	**6.51**	**20.03**

a The name of the QTL is defined by the chromosome number.

b The traits-years-treatments of QTL is a composite of the influenced trait: RDW, root dry weight; SDW, shoot dry weight; TDW, total dry weight; R/S, root to shoot ratio; PC, P concentration; Pup, phosphorus uptake; PUE, phosphorus use efficiency; BY, biomass yield; PN, net photosynthetic rate; Tr, transpiration rate; and Ci, intercellular carbon dioxide concentration; Co, stomatal conductance; and CC, chlorophyll content; followed by the environments, treatments and growth stages. NP denotes a QTL underlying the influenced trait at normal P condition, and LP denotes a QTL at low P condition.

c Chr indicates chromosome.

d Interval indicates confidence interval between two SLAF markers.

e Position indicates the interval of physical distance in soybean genome.

f LOD indicates the average logarithm of odds score.

g PVE^2^ indicates the average phenotypic variance explained by related QTL. Major QTLs are shown in bold.

By further analyzing these 29 loci, we found that 11 loci could be repeatedly detected more than five times across traits, treatments or environments, and these 11 loci were subsequently defined as the major QTLs (Figure 3, Table 3, Figure S1). These QTLs might represent a majority of genetic basis of PE and photosynthesis and thus would be focused on in the subsequent analyses. As shown in Table 3, the 11 QTLs (*q2-1, q3-1, q4-3, q8-2, q11-2, q12-1, q13, q14-2, q15-2, q18-1*, and *q19-2*) were distributed on chromosomes 2, 3, 4, 8, 11, 12, 13, 14, 15, 18, and 19. The average LOD score of these QTLs ranged from 3.19 to 8.36, and the average phenotypic variance explained by individual locus ranged from 7.18 to 22.00% (Table 3, Table S3). In addition, comparative analyses showed that seven major QTLs (*q3-1, q4-3, q8-2, q13, q14-2, q15-2*, and *q19-2*) identified in this study were co-localized with previous identified PE related QTLs (Zhang et al., 2009, 2016), including the QTL harboring PE key genes, *GmACP1* (Zhang et al., 2014). These seven QTLs, which were identified across environments and growth stages, might play important roles for P efficiency during both soybean seedling and reproductive stages. For example, the major QTL, *q8-2*, underlying PC, Pup, PUE, and BY was stably identified across traits and environments. This QTL located on 1.9 Mb on chromosome 8, where the acid phosphatase encoding gene *GmACP1* located, with LOD scores ranging from 3.03 to 4.24, explaining 6.68–9.50% of the phenotypic variation. The co-localization of *GmACP1* with *q8-2* provided a strong evidence showing the accuracy of the mapping results in the present study.

Given that both PE and photosynthetic related traits are complex traits, epistatic effects may exist between different QTLs. Thus, in addition to these additive QTLs, we also analyzed the significant epistatic loci for both traits across treatments using the ICIM-EPI modules of QTL IciMapping. Among the 78 trait combinations (PE and photosynthetic related trait measured under two P treatments across environments), a total of 35 pairs of QTLs, which had epistatic interactions with each other, were identified on almost all 20 chromosomes (except for 12). These QTLs explained 10.7–53.9% of phenotypic variance (Figure 3, Table S4). Among these epistatic loci, 11 pairs were identified for the both related traits at NP condition, while 25 pairs were identified at LP condition, suggesting that low P stress may induce the expression of epistatic genes underlying epistatic loci. Three pleiotropic epistatic QTLs were detected between chromosomes 1 and 4, 1, and 6, and 4 and 7 across traits and environments.

### Determination of co-localized QTLs and trait-specific QTLs

Of 29 loci, 12 (~40%, *q2-1, q2-2, q3-1, q3-2, q4-3, q5, q8-1, q13, q14-2, q15-1, q15-2*, and *q19-2*) were associated with both PE and photosynthetic related traits, which was consistent with the high correlation between both traits (Figure 2, Table S2). Among 12 loci, *q19-2* ranked the largest QTL cluster harboring 28 QTLs associated with 22 PE and six photosynthetic related traits across environments, including PC, Pup, PUE, biomass, CC, and Tr. The average LOD score of this locus was 6.51 and these QTLs could explain 20.03% of average phenotypic variance (Figure 3, Table 3, Table S3). Similarly, another QTL, *q15-2*, was associated with 14 PE and four photosynthetic related traits across environments, including PC, Pup, PUE, biomass, Ci, Co, and Tr. The average LOD score of this locus was 6.04 (ranged from 2.53 to 17.61), and these QTLs could explain 6.61–58.91% of phenotypic variance (Table S3). Therefore, these QTLs, such as *q15-2* and *q19-2*, with high LOD value and phenotypic variance explanation may be important loci contributing to PE and photosynthesis. In addition, three LP-specific QTLs, *q2-1, q5*, and *q8-1* associated with both traits could be detected only under LP condition, suggesting that some pleiotropic genes within three LP-specific QTLs were LP-specific inducible and regulate PE and photosynthesis simultaneously. Therefore, co-localization of a number of QTLs associated with both PE and photosynthesis traits suggested the significant genetic contribution of PE to photosynthetic traits in soybean.

Interestingly, we identified two photosynthetic-specific loci, *q1* and *q12-1*, on chromosome 1 and 12 under both P conditions. These two QTLs were not linked to any of the PE traits, suggesting that this QTLs may not affected by low P stress. The average LOD score of the loci, *q12-1*, could be up to 7.37 (ranged from 3.1 to 12.5), and these QTLs could explain 7.2–44.4% of phenotypic variance. Moreover, all the favorable alleles of *q12-1* were from the high biological-yielding parent (Bogao). These results suggest that *q12-1* could be effectively used in soybean breeding programs for high photosynthetic efficiency improvement. In addition, another two specific QTLs, *q10*, and *q18-1*, were detected associated with CC only under LP condition across environments, which suggested that the genes underlying these QTLs might be more favorably induced by LP stress. The QTL, *q18-1*, with the largest average LOD score (8.4) can explain 7.8–39.3% of the phenotypic variance. Deep characterization of these photosynthetic specific QTLs may increase our understanding of the genetic basis of photosynthetic traits.

### Prediction and preliminary validation of candidate genes

In our present study, the average size of the confidence intervals was ~1.7 Mb in physical distance based on the high-density genetic map (Table 3). These intervals were relatively narrower than those identified in previous studies (Zhang et al., 2009) in which the average size of these related QTL intervals is ~10 Mb based on a linkage map constructed by 306 SSR markers. To identify candidate genes affecting each trait, annotated genes within five promising genomic regions (*q12-1, q14-2, q15-2, q18-1*, and *q19-2*) with relatively large *r*^2^ and LOD score and stably expressing across environments (Table 3), were investigated based on annotation of soybean reference genome W82.a2.v1. A summary of the candidate genes for each promising region was shown in Table S5. For example, *q19-2* that was previously identified associated with P efficiency (Zhang et al., 2009, 2014) was located at a ~4-Mb region on chromosome 19. Several predicted genes encoding purple acid phosphatase (*Glyma.19G193900*), NPH3 protein (*Glyma.19G207900*), NADPH dehydrogenase (*Glyma.19G254700*), photosystem I P subunit (*Glyma.19G260600*), Chlorophyll A binding protein (*Glyma.19G261400*), and photosystem II reaction center PsbP family protein (*Glyma.19G227400*) were predicted in this interval, and might be involved in the phosphate and photosynthetic metabolic process. Among these genes, *Glyma.19G193900* was previously up-regulated significantly (more than 30-fold) in soybean leaves under low P conditions compared with in control by transcriptome analysis (Zhang et al., 2016, in review).

**Figure 4:**
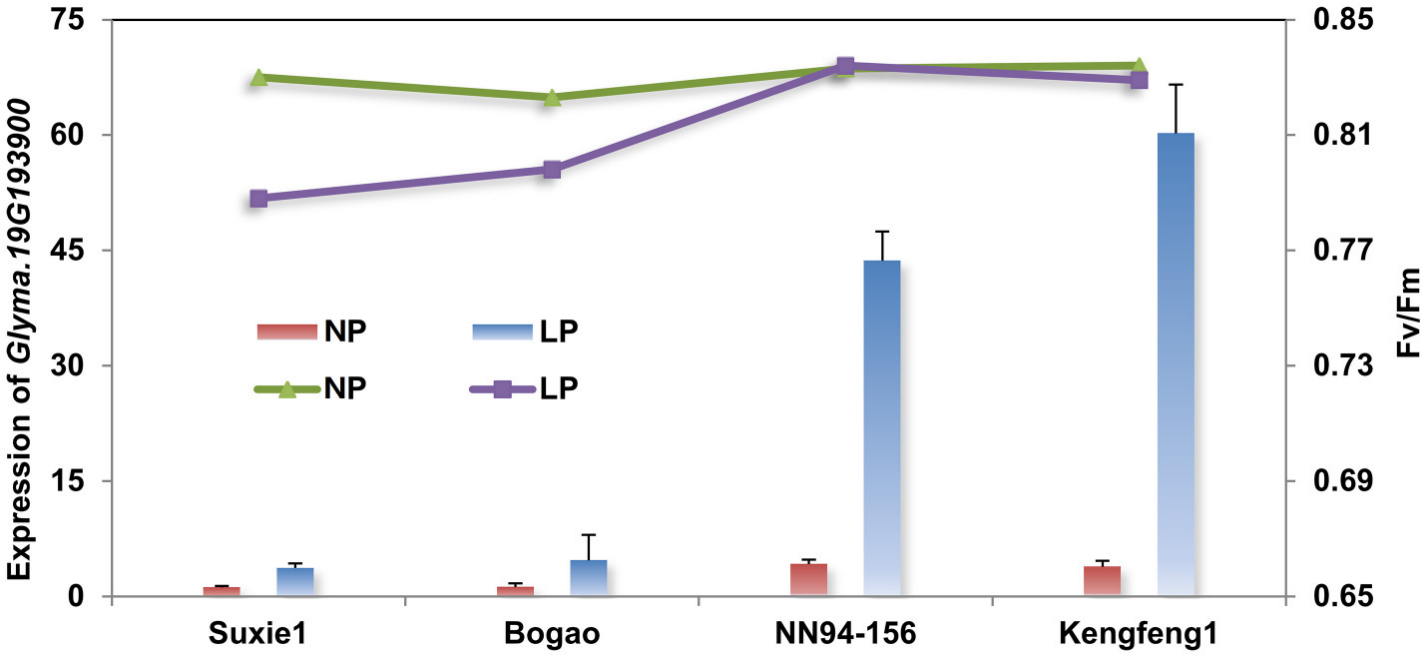
***q*RT-PCR analyses of the candidate gene (*Glyma.19G193900*) in four representative accessions with different P efficiency**. The primary Y-axis denotes the gene expression levels, the secondary Y-axis denotes the chlorophyll fluorescence parameter Fv/Fm (maximum quantum efficiency of photosystem II (PSII) photochemistry). NP and LP denote normal P and low P conditions. Suxie1 and Bogao are P-inefficient genotypes and NN94-156 and Kengfeng1are P-efficient genotypes.

In order to investigate the involvement of gene *Glyma.19G193900* in soybean P regulation, a quantitative real-time PCR (qRT-PCR) analysis was performed using four representative accessions (contain two P-efficient genotypes, Nannong94-156 and Kegeng1, and two P-inefficient genotypes, Sxie1 and Bogao) at 7 days after low P stress. The result showed that expression level of *Glyma.19G193900* was increased by 40- to 60-fold in the both P-efficient genotypes, of which photosynthetic related traits, such as Fv/Fm (maximum quantum efficiency of photosystem II (PSII) photochemistry) and Pn, have not been significantly affected by low P stress (Figure 4). By contrast, the expression of *Glyma.19G193900* was not significant affected in both P-inefficient genotypes, and the photosynthetic related traits have been decreased significantly under low P stress (Figure 4).

For the interesting major loci, *q12-1*, which were detected only underlying nine different photosynthetic traits under both P conditions, and located in a region of 1.6-Mb physical region in reference genome, containing ~180 putative predicted genes. Enriched GO categories revealed that four predicted genes encoding an ATP synthase (*Glyma.12G056400*), two NADPH dehydrogenase (*Glyma.12G059100* and *Glyma.12G059400*) and a ribulose bisphosphate (RubP) carboxylase (*Glyma.12G061600*) in this interval might be involved in photosynthesis process (Table S4). Moreover, our previous transcriptome analysis results showed that the expression level of NADH dehydrogenase (*Glyma.12G059100*) increased significantly in soybean leaves under low P conditions compared with control (Zhang et al., 2016, in review). The gene *Glyma.12G061600* was also regarded as a candidate for photosynthesis, as expression of RubP gene was reported to be key regulation points for photosynthesis under different environmental stress conditions (Crafts-Brandner and Salvucci, 2000; Portis, 2003).

Another important locus, *q18-1*, associated with photosynthetic traits only under LP condition was mapped in an approximate 0.85 Mb genomic region flanked by markers Marker2217116 and Marker2237156 on chromosome 18. This region contains 46 annotated genes encoding photosystem II (*Glyma.18G028400*), Rubisco activase (RCA, *Glyma.18G036400*) and three NADPH dehydrogenases (*Glyma.18G035100, Glyma.18G035200*, and *Glyma.18G036100*; Table S5). It is noteworthy that the candidate gene, *Glyma.18G036400* was consistent with a photosynthetic crucial gene *GmRCA*β, which has been previously reported to be involved in catalyzing the activation of Rubisco, a key enzyme regulating photosynthesis (Yin et al., 2010b). The *GmRCA*β gene was localized to the correct genomic regions, suggesting the high accuracy of the results in the present study. In QTL, *q15-2*, a comprehensive analysis of this region predicted 190 putative genes. Three of these genes encoding wall-associated kinase family protein (*Glyma.15G133400*), phototropic-responsive NPH3 family protein (*Glyma.15G121700*), and copper transport protein family (*Glyma.15G122200*) were regarded as candidates related to low P stress tolerance and photosynthesis based on Blastp querying in the protein database (Table S5).

## Discussion

Crop growth and productivity relies on photosynthesis. Given the important function of photosynthesis, crop breeders are turning their attention to improve photosynthesis capability to increase crop yield with the sustainable use of natural resources. Previous attempts to enhance nitrogen, phosphorus and water use efficiency have been achieved by genetic improvements of photosynthetic related traits in crops (Pelleschi et al., 2006; Guo et al., 2008; Kiani et al., 2008; Zhao et al., 2008; Cai et al., 2012). From a sustainable development perspective, efficient use of P in photosynthesis is a potentially important determinant for sustainable crop production (Veneklaas et al., 2012). On the other hand, selecting P-efficient genotypes in breeding programs has the potential to enhance photosynthesis capability. However, this type of breeding practice is limited because of a lack of knowledge on genetic relationship between PE and photosynthesis and the corresponding genomic regions for targeted genetic manipulation. In this study, a soybean RIL population possessing a large variation in both PE and photosynthetic related traits was used to uncover the significant genetic associations between PE and photosynthesis, and to identify related QTLs and candidate genes. These findings allow the establishment of a PE-based approach for genetically improving photosynthesis in soybean.

### Mapping population and genetic variation of PE and photosynthetic related traits

The selection of mapping populations is important for constructing a high-density map and further dissection of mapped QTLs. The phenotypes investigated in this population, derived from a cross between Nannong94-156 and Bogao, and exhibited significant variation in plant height, biological yield, number of main stems and 100-seed weight, responses to low P stress and photosynthetic related traits. Thus, this RIL population has been used to identify many useful traits mentioned above. For example, this population have been used to mapped QTLs for P efficiency, biological yield, apparent harvest index, flowering time, brachytic stem and chlorophyll content (Cui and Yu, 2007; Cui et al., 2007a,b, 2008; Zhang et al., 2009, 2013, 2014). Recently, we used the same population to construct a high-density genetic map with 6159 SNP markers, and identified a novel QTLs underlying P efficiency based on this map (Zhang et al., 2016). In addition, the two parents differed considerably for the chlorophyll and chlorophyll fluorescence parameters when grown in field and greenhouse conditions (Hao et al., 2012). Thus, this population is suitable for investigation of both PE and photosynthetic related traits, and the genetic relationship between them.

In the present study, the two parents differed considerably for the chlorophyll content (CC), net photosynthetic rate (Pn), and transpiration rate (Tr) when grown under NP and LP conditions (Table 1), indicating that photosynthesis was affected significantly by low P stress. The large phenotypical variation for the agronomically important traits between the parental lines and within the derived population allows the effective dissection of the genetic basis and identification of genomic regions underlying these important traits. Compared with the biomass and P acquisition in parent Bogao was higher than in parent Nannong94-156 in different P levels and trials, which was consistent with the fact that Bogao was a high biological-yielding soybean variety. However, higher R/S and PUE in Nannong94-156 than Bogao through all our tests suggest that Nannong94-156 was a low P stress tolerant variety, which was also previously identified (Zhang et al., 2009). As expected, QTL analysis revealed the additive effect of 80% of QTLs associated with PE was derived from the donor allele of Nannong94-156, while about half of the additive effect for photosynthetic QTLs was derived from the donor allele of Bogao. Consequently, this established RIL population from two contrasted lines differing in PE and photosynthetic related traits was suitable for determining the genetic relationship between the both traits, and identifying favorable alleles from Nannong94-156 for PE and photosynthetic capacity improvement.

### Genetic relationship between PE and photosynthetic related traits in soybean

Crop growth and productivity relies on photosynthesis, and the photosynthetic processes are largely relying on P (Veneklaas et al., 2012). However, due to the complexity of PE and photosynthetic related traits, strong QTL-environment interaction, possible epistatic effects and small explanation of loci, the knowledge on the both traits are still incomplete. Consequently, the genetic relationship between PE and photosynthetic related traits are not clear. In photosynthetic processes phosphorous may limit the transformation of atmospheric CO_2_ into organic carbon, and in biochemical processes atmospheric CO_2_ may limit the mobilization of phosphorous (Föllmi et al., 2004), suggesting that the phosphorous and carbon cycles are characterized by interactions and resulting feedback mechanisms, which show the close relationship between PE and photosynthesis. The importance of photosynthesis and existence of a close relationship between PE and photosynthetic traits makes it essential for breeders to consider photosynthetic capacity as a selection criterion in soybean P-efficient breeding programs, or to say efficient use of P in photosynthesis. Given that both soybean PE and photosynthetic related traits are complex traits and controlled by multiple genes, accurate evaluation of them in field is extremely challenging and labor-consuming, and strongly affected by environments. Therefore, in this study, we have adopted hydroponic and pot culture experiments to investigate the phenotypic traits in seedling and flowering podding stage, respectively.

In the present study, the phenotypical correlation analysis and PCA showed that photosynthetic related traits significantly associated with PE (*r* = 0.14–0.27; Figure 2, Table S2). Importantly, photosynthetic related traits had a significantly correlation with PC, Pup and PUE (*r* = 0.15–0.31), suggesting that P acquisition and utilization are likely related to the capacity of photosynthesis. Among photosynthetic related traits, particularly Pn and Tr under LP conditions, seem to be more relevant to PC (*r* = 0.31; Table S2). Besides PE traits, a similar significantly correlation between photosynthetic related traits and biomass were also found across environments, suggesting that photosynthesis may to large extent determine the soybean productivity. Therefore, the selection of P-efficient genotypes is a promising approach to optimize soybean photosynthetic capacity, and even the yield. Under pot conditions, a significant correlation (*r* = 0.30–0.43) was also found between BY and CC at R6 stage using the same population in this study. In a previous study (Cai et al., 2012), the significant relationships has been reported between maize photosynthetic related traits and grain yield under LP condition, which further implicate the essential function of photosynthesis on adaptation of plants to abiotic stress in general.

Overlapped QTLs as revealed in this study further supports the closely genetic relationship between PE and photosynthetic related traits (Figure 3, Table 3, Table S3). A large proportion of PE-QTLs (40%) are associated with QTLs for photosynthetic related traits (Table S3), and several major QTL clusters contained QTLs for both PE and photosynthetic related traits (Figure 3). Moreover, favorable alleles of these most QTL clusters coming from the parent Nannong94-156, which was a high P efficiency soybean variety and has a better yield than Bogao under low P stress. In addition, in a previous study, most of QTLs for chlorophyll content were detected in the same soybean RIL population also had favorable effects of alleles from the parent (Nannong94-156) with higher P efficiency (Cui and Yu, 2007). It is worth noting that the relationship between PE and photosynthetic related traits at QTL clusters may correspond to control of pleiotropic genes or to different closely linked genes. Nevertheless, the presence of significant phenotypic correlation and co-localized common QTLs provide the solid genetic basis for establishing the association between PE and photosynthetic related traits.

### Important QTLs and potential candidate genes for both traits of interest

In this study, the coincidence of QTL for PE and photosynthetic related traits provides clues on their genetic association. More importantly, identification of the optimal alleles among these QTLs can help soybean breeders use high-PE and high-photosynthetic efficiency cultivars via a marker-assisted selection approach. Several important QTLs were identified in which QTLs for PE and photosynthetic related traits coincided or specific (Figure 3). For example, the *q19-2* was co-localized or adjacent QTL regions, which were identified to be associated with PAE and PUE (Zhang et al., 2009) and chlorophyll content (Cui and Yu, 2007), suggesting the presence of important genes involved in regulating both traits. A putative gene, encoding a purple acid phosphatase (*Glyma.19G193900*) in this region was regarded as a possible candidate, as it is generally believed to be important for P acquisition and utilization in various plant species (Duff et al., 1994), such as in *Arabidopsis thaliana* (Li et al., 2002), soybean (Zhang et al., 2014), tomato (Baldwin et al., 2001), and white lupin (Wasaki et al., 2009). Moreover, previous studies have shown that the phosphatases involved in the regulation of photosynthesis (Chen et al., 2005; Rochaix et al., 2012). The significantly increased expression level of *Glyma.19G193900* also implicated its roles in responding to low P stress (Figure 4). Thus, *Glyma.19G193900* was putatively considered to be involved in regulating the both traits, and deserving to be further experimentally verified. In addition, we also found several photosynthetic related genes, including NADPH dehydrogenase, Chlorophyll A binding protein, photosystem I P subunit, photosystem II reaction center PsbP family protein and NPH3 protein within this region, which might be involved in the phosphate and photosynthetic metabolic process as previous described (Baldwin et al., 2001; Rochaix et al., 2012; Zhang et al., 2014).

An important photosynthetic-specific QTL, *q12-1*, were detected on chromosome 12 under both NP and LP conditions, suggesting that this QTL's expression is less affected by low P stress. Thus, *q12-1* could be a promising candidate locus for further high photosynthetic efficiency study. Development of near isogenic lines based on this region could be the best approach to fine map this QTL without the interference of other P efficiency genes. In addition, the role of Ribulose-bisphosphate in photosynthesis have been previously identified (Yin et al., 2010b), thus (*Glyma.12G061600*) could be one of the candidate genes underlying this loci. Correlation of Ribulose-bisphosphate gene expression levels with photosynthetic traits indicates that RCA genes could play an important role in regulating soybean photosynthetic capacity and seed yield (Yin et al., 2010b).

In contrast to *q12-1*, the LP specific-QTLs (*q18-1*) on chromosome 18 were detected for photosynthetic related traits only under LP conditions, suggesting that this QTL's expression may be inducible by low P stress. When QTLs for PC and Pup was added to the analysis as covariates, the LOD and additive effect of *q18-1* were all decreased (data not shown), suggesting that these photosynthetic traits were P dependent. On the other hand, it may be a crucial response mechanism of tolerance to low P stress by slowing photosynthesis. Under low P stress, reduction of chlorophyll content thus slowed the photosynthesis rate and transpiration rate, which may be beneficial for plants to escape from stress at the expense of reducing yield potential. In addition, in this region, it is noteworthy that the *q18-1* underlying gene, *Glyma.18G036400*, which encoding a RCA gene was corresponded to the photosynthetic key gene (*GmRCA*β), with the largest average LOD score (8.4) and explain 7.8–39.3% of the phenotypic variance. Correlation of gene expression levels with photosynthetic related traits indicates that *GmRCA*β gene could play an important role in regulating soybean photosynthetic capacity and seed yield (Yin et al., 2010b). Further study showed that regulating expression levels of Rubisco activase (*GmRCA*β) gene to optimize Rubisco activation may provide an approach to enhance plant productivity (Chao et al., 2014). Thus, co-localization of *GmRCA*β with *q18-1* and the narrowed QTL regions provided a strong evidence showing the high accuracy of the mapping strategy in the present study. Deep dissection of these major QTLs and promising candidate genes for both traits could provide greater understanding of the genetic basis of PE and photosynthetic traits. Although the promising genes were provided within these loci, more studies such as overexpression or knock-out of these genes are needed to uncover the molecular genetic mechanism underlying PE and photosynthesis.

In summary, despite the highly complex nature of PE and photosynthetic related traits, our study shows a close genetic relationship and of major QTLs that coincide for both traits, implicating that genetic improvement for PE and photosynthetic efficiency in soybean could be achieved by introducing one QTL. Previous studies have investigated these traits at the physiological level, the findings presented here provide a better knowledge of the genetic factors to produce P-efficient and photosynthetic-efficient soybean genotypes. The important major QTL, *q12-1*, associated with photosynthetic related traits was less affected by low P stress, this locus and candidate RCA genes can be effectively used in breeding programs for photosynthesis improvement. The markers linked to these valuable QTLs could be further tested for marker-assisted selection of desired genotypes with improved PE and photosynthetic efficiency. Because it is impossible to distinguish a pleiotropy or linkage between adjacent loci at the current level of QTL clustering, fine mapping and positional cloning are required to identify the underlying gene or genes to further to genetically improving soybean PE and photosynthetic capacity. This is the first paper using simultaneously measured PE and photosynthetic traits data to study intensively the genetic differences in photosynthesis under different P levels across environments.

## Author contributions

DZ, HL conceived and designed the experiments. HL, YY, and SC performed the experiments, including hydroponics experiments and pot experiments. HL and YY performed data analyses and QTL mapping. HL, HZ, and DZ wrote the manuscript. DY provided materials. All authors have read and approved the final version of the manuscript to be published.

### Conflict of interest statement

The authors declare that the research was conducted in the absence of any commercial or financial relationships that could be construed as a potential conflict of interest.

## Supplementary material

Table S1 **Descriptive statistical results for PE and photosynthetic related traits in soybean recombinant inbred lines (RILs) across treatments and experiments**. Statistics for RDW, root dry weight; SDW, shoot dry weight; TDW, total dry weight; R/S, root to shoot ratio; PC, P concentration; Pup, phosphorus uptake; PUE, phosphorus use efficiency; BY, biomass yield; PN, net photosynthetic rate; Tr, transpiration rate ; and Ci, intercellular carbon dioxide concentration; Co, stomatal conductance; and CC, chlorophyll content; followed by the treatments and environments. See Table 1 for an explanation of trait abbreviations and their units.

Table S2 **Pearson's correlation coefficients (r) between all investigated traits of RILs grown under normal P (NP) and low P (LP) levels**.

Table S3 **Summary of detected QTLs for all investigated traits in different treatments, environments, and growth stages**.

Table S4 **Epistatic loci f or investigated traits under normal P (NP) and low P (LP) conditions**.

Table S5 **Predicted candidate genes associated with PE and photosynthetic related traits in QTL regions**.

Figure S1 **Quantitative trait loci (QTL) for PE and photosynthetic related traits at normal P (NP) and low P (LP) conditions**. The designation on the left/right is genetic distance (cM) and marker name. The name of each QTL, is a composite of the influenced trait: RDW, root dry weight; SDW, shoot dry weight; TDW, total dry weight; R/S, root to shoot ratio; PC, P concentration; Pup, phosphorus uptake; PUE, phosphorus use efficiency; BY, biomass yield; PN, net photosynthetic rate; Tr; transpiration rate; and Ci, intercellular carbon dioxide concentration; Co, stomatal conductance; and CC, chlorophyll content; followed by the treatments, environments, and growth stages. See Table 1 for an explanation of trait abbreviations and their units.

Figure S2 **QTLs indicated by the number of significant QTLs plotted for 20 chromosomes (Chr.01-Chr.20) under normal P (NP) and low P (LP) conditions**. The gray dotted vertical lines separate the 20 chromosomes.

## References

[B1] Ao J. Fu J. Tian J. Yan X. Liao H. (2010). Genetic variability for root morph-architecture traits and root growth dynamics as related to phosphorus efficiency in soybean. Funct. Plant Biol. 37, 304–312. 10.1071/FP09215

[B2] Baldwin J. C. Karthikeyan A. S. Raghothama K. G. (2001). *LEPS2*, a phosphorus starvation-induced novel acid phosphatase from tomato. Plant Physiol. 125, 728–737. 10.1104/pp.125.2.728 11161030 PMC64874

[B3] Balemi T. Negisho K. (2012). Management of soil phosphorus and plant adaptation mechanisms to phosphorus stress for sustainable crop production: a review. J. Soil Sci. Plant Nutr. 12, 547–562. 10.4067/s0718-95162012005000015

[B4] Brooks A. Woo K. Wong S. (1988). Effects of phosphorus nutrition on the response of photosynthesis to CO_2_ and O_2_, activation of ribulose bisphosphate carboxylase and amounts of ribulose bisphosphate and 3-phosphoglycerate in spinach leaves. Photosyn. Res. 15, 133–141. 10.1007/BF00035257 24430858

[B5] Cai H. Chu Q. Yuan L. Liu J. Chen X. Chen F. (2012). Identification of quantitative trait loci for leaf area and chlorophyll content in maize (*Zea mays*) under low nitrogen and low phosphorus supply. Mol. Breed. 30, 251–266. 10.1007/s11032-011-9615-5

[B6] Chao M. Yin Z. Hao D. Zhang J. Song H. Ning A.. (2014). Variation in Rubisco activase (*RCA*β) gene promoters and expression in soybean [*Glycine max* (L) Merr]. J. Exp. Bot. 65, 47–59. 10.1093/jxb/ert346 24170743 PMC3883283

[B7] Chen S. Hajirezaei M. Peisker M. Tschiersch H. Sonnewald U. Börnke F. (2005). Decreased sucrose-6-phosphate phosphatase level in transgenic tobacco inhibits photosynthesis, alters carbohydrate partitioning, and reduces growth. Planta 221, 479–492. 10.1007/s00425-004-1458-4 15657716

[B8] Cordell D. White S. (2011). Peak phosphorus: clarifying the key issues of a vigorous debate about long-term phosphorus security. Sustainability 3, 2027–2049. 10.3390/su3102027

[B9] Crafts-Brandner S. J. Salvucci M. E. (2000). Rubisco activase constrains the photosynthetic potential of leaves at high temperature and CO2. Proc. Natl. Acad. Sci. U.S.A. 97, 13430–13435. 10.1073/pnas.230451497 11069297 PMC27241

[B10] Cui S. Geng L. Meng Q. Yu D. (2007a). QTL mapping of phosphorus deficiency tolerance in soybean (*Glycine max* L.) during seedling stage. Soybean Sci. 33, 378–383. 10.3321/j.issn:0496-3490.2007.03.005

[B11] Cui S. He X. Fu S. Meng Q. Gai J. Yu D. (2008). Genetic dissection of the relationship of apparent biological yield and apparent harvest index with seed yield and yield related traits in soybean. Crop Pasture Sci. 59, 86–93. 10.1071/AR07068

[B12] Cui S. Meng Q. Gai J. Yu D. (2007b). Gene mapping of brachytic stem and its effects on yield-related traits in soybean. Crop Pasture Sci. 58, 774–779. 10.1071/AR06358

[B13] Cui S. Yu D. (2007). QTL mapping of chlorophyll content at various growing stages and its relationship with yield in soybean [*Glycine max* (L.) Merr.]. Soybean Sci. 33, 744–750. 10.3321/j.issn:0496-3490.2007.05.008

[B14] Du Z. Zhou X. Ling Y. Zhang Z. Su Z. (2010). agriGO: a GO analysis toolkit for the agricultural community. Nucleic Acids Res. 38, W64–W70. 10.1093/nar/gkq310 20435677 PMC2896167

[B15] Duff S. M. Sarath G. Plaxton W. C. (1994). The role of acid phosphatases in plant phosphorus metabolism. Physiol. Plant. 90, 791–800. 10.1111/j.1399-3054.1994.tb02539.x

[B16] Föllmi K. B. Tamburini F. Hosein R. Van de Schootbrugge B. Arn K. Rambeau C. (2004). Phosphorus, a servant faithful to Gaia? Biosphere remediation rather than regulation, in Scientists Debate Gaia: The Next Century, eds Schneider S. H. Miller J. R. Crist E. Boston P. J. (Cambridge, MA: MIT Press), 79–92.

[B17] Fredeen A. L. Raab T. K. Rao I. M. Terry N. (1990). Effects of phosphorus nutrition on photosynthesis in *Glycine max* (L) Merr. Planta 181, 399–405. 10.1007/BF00195894 24196818

[B18] Gaxiola R. A. Sanchez C. A. Paez-Valencia J. Ayre B. G. Elser J. J. (2012). Genetic manipulation of a “vacuolar” H+-PPase: from salt tolerance to yield enhancement under phosphorus-deficient soils. Plant Physiol. 159, 3–11. 10.1104/pp.112.195701 22434041 PMC3375966

[B19] Gowin S. (1997). Phosphorus and potassium effects on soybeans, in Horticultural Crops Plant Nutrition Series, ed Storey J. B. (College Station, TX: Department of Horticultural Science, Texas A&M University), 44–51.

[B20] Guo P. Baum M. Varshney R. K. Graner A. Grando S. Ceccarelli S. (2008). QTLs for chlorophyll and chlorophyll fluorescence parameters in barley under post-flowering drought. Euphytica 163, 203–214. 10.1007/s10681-007-9629-6

[B21] Hao D. Chao M. Yin Z. Yu D. (2012). Genome-wide association analysis detecting significant single nucleotide polymorphisms for chlorophyll and chlorophyll fluorescence parameters in soybean (*Glycine max*) landraces. Euphytica 186, 919–931. 10.1007/s10681-012-0697-x

[B22] Høghjensen H. Schjoerring J. Soussana J. (2002). The influence of phosphorus deficiency on growth and nitrogen fixation of white clover plants. Ann. Bot. 90, 745–753. 10.1093/aob/mcf260 12451030 PMC4240371

[B23] Kiani S. P. Maury P. Sarrafi A. Grieu P. (2008). QTL analysis of chlorophyll fluorescence parameters in sunflower (*Helianthus annuus* L.) under well-watered and water-stressed conditions. Plant Sci. 175, 565–573. 10.1016/j.plantsci.2008.06.002

[B24] Li D. Zhu H. Liu K. Liu X. Leggewie G. Udvardi M.. (2002). Purple acid phosphatases of *Arabidopsis thaliana* comparative analysis and differential regulation by phosphate deprivation. J. Biol. Chem. 277, 27772–27781. 10.1074/jbc.M204183200 12021284

[B25] Li Y. Luo A. Hassan M. Wei X. (2006). Effect of phosphorus deficiency on leaf photosynthesis and carbohydrates partitioning in two rice genotypes with contrasting low phosphorus susceptibility. Rice Sci. 13, 283–290.

[B26] Li Y. Wang Y. Tong Y. Gao J. Zhang J. Chen S. (2005). QTL mapping of phosphorus deficiency tolerance in soybean (*Glycine max* L. Merr.). Euphytica 142, 137–142. 10.1007/s10681-005-1192-4

[B27] Liang Q. Cheng X. Mei M. Yan X. Liao H. (2010). QTL analysis of root traits as related to phosphorus efficiency in soybean. Ann. Bot. 106, 223–234. 10.1093/aob/mcq097 20472699 PMC2889805

[B28] Livak K. J. Schmittgen T. D. (2001). Analysis of relative gene expression data using real-time quantitative PCR and the 2−ΔΔCT method. Methods 25, 402–408. 10.1006/meth.2001.1262 11846609

[B29] Murphy J. Riley J. P. (1962). A modified single solution method for the determination of phosphate in natural waters. Anal. Chim. Acta 27, 31–36. 10.1016/S0003-2670(00)88444-5

[B30] Nielsen K. L. Eshel A. Lynch J. P. (2001). The effect of phosphorus availability on the carbon economy of contrasting common bean (*Phaseolus vulgaris* L.) genotypes. J. Exp. Bot. 52, 329–339. 10.1093/jexbot/52.355.329 11283178

[B31] Öckinger J. Serrano-Fernández P. Möller S. Ibrahim S. M. Olsson T. Jagodic M. (2006). Definition of a 1.06-Mb region linked to neuroinflammation in humans, rats and mice. Genetics 173, 1539–1545. 10.1534/genetics.106.057406 16624898 PMC1526695

[B32] Pelleschi S. Leonardi A. Rocher J.-P. Cornic G. De Vienne D. Thevenot C. (2006). Analysis of the relationships between growth, photosynthesis and carbohydrate metabolism using quantitative trait loci (QTLs) in young maize plants subjected to water deprivation. Mol. Breed. 17, 21–39. 10.1007/s11032-005-1031-2

[B33] Portis A. R Jr.. (2003). Rubisco activase-Rubisco's catalytic chaperone. Photosynth. Res. 75, 11–27. 10.1023/A:1022458108678 16245090

[B34] Rao I. M. Terry N. (1995). Leaf phosphate status, photosynthesis, and carbon partitioning in sugar beet (IV. Changes with time following increased supply of phosphate to low-phosphate plants). Plant Physiol. 107, 1313–1321. 12228438 10.1104/pp.107.4.1313 PMC157266

[B35] Rochaix J.-D. Lemeille S. Shapiguzov A. Samol I. Fucile G. Willig A.. (2012). Protein kinases and phosphatases involved in the acclimation of the photosynthetic apparatus to a changing light environment. Philos. Trans. R. Soc. Lond. B Biol. Sci. 367, 3466–3474. 10.1098/rstb.2012.0064 23148273 PMC3497069

[B36] Singh S. K. Badgujar G. Reddy V. R. Fleisher D. H. Bunce J. A. (2013). Carbon dioxide diffusion across stomata and mesophyll and photo-biochemical processes as affected by growth CO2 and phosphorus nutrition in cotton. J. Plant Physiol. 170, 801–813. 10.1016/j.jplph.2013.01.001 23384758

[B37] Swanson-Wagner R. DeCook R. Jia Y. Bancroft T. Ji T. Zhao X.. (2009). Paternal dominance of trans-eQTL influences gene expression patterns in maize hybrids. Science 326, 1118–1120. 10.1126/science.1178294 19965432

[B38] Vaccari D. A. (2009). Phosphorus: a looming crisis. Sci. Am. 300, 54–59. 10.1038/scientificamerican0609-54 19485089

[B39] Van Vuuren D. P. Bouwman A. Beusen A. (2010). Phosphorus demand for the 1970-2100 period: a scenario analysis of resource depletion. Glob. Environ. Change 20, 428–439. 10.1016/j.gloenvcha.2010.04.004

[B40] Venables W. N. Smith D. M. (2005). The R Development Core Team. An Introduction to R. Notes on R: A Programming Environment for Data Analysis and Graphics. Bristol: Network Theory Ltd.

[B41] Veneklaas E. J. Lambers H. Bragg J. Finnegan P. M. Lovelock C. E. Plaxton W. C.. (2012). Opportunities for improving phosphorus - use efficiency in crop plants. New Phytol. 195, 306–320. 10.1111/j.1469-8137.2012.04190.x 22691045

[B42] Visscher P. Thompson R. Haley C. (1996). Confidence intervals in QTL mapping by bootstrapping. Genetics 143, 1013–1020. 8725246 10.1093/genetics/143.2.1013 PMC1207319

[B43] Wang X. Wurmser C. Pausch H. Jung S. Reinhardt F. Tetens J.. (2012). Identification and dissection of four major QTL affecting milk fat content in the german holstein-friesian population. PLoS ONE 7:e40711. 10.1371/journal.pone.0040711 22792397 PMC3394711

[B44] Wang Z. Cui Y. Chen Y. Zhang D. Liang Y. Zhang D.. (2014). Comparative genetic mapping and genomic region collinearity analysis of the powdery mildew resistance gene *Pm41*. Theor. Appl. Genet. 127, 1741–1751. 10.1007/s00122-014-2336-5 24906815

[B45] Wasaki J. Maruyama H. Tanaka M. Yamamura T. Dateki H. Shinano T. (2009). Overexpression of the *LASAP2* gene for secretory acid phosphatase in white lupin improves the phosphorus uptake and growth of tobacco plants. Soil Sci. Plant Nutr. 55, 107–113. 10.1111/j.1747-0765.2008.00329.x

[B46] Yin Z. Meng F. Song H. He X. Xu X. Yu D. (2010a). Mapping quantitative trait loci associated with chlorophyll a fluorescence parameters in soybean (*Glycine max* (L) Merr). Planta 231, 875–885. 10.1007/s00425-009-1094-0 20183920

[B47] Yin Z. Meng F. Song H. Wang X. Xu X. Yu D. (2010b). Expression quantitative trait loci analysis of two genes encoding rubisco activase in soybean. Plant Physiol. 152, 1625–1637. 10.1104/pp.109.148312 20032079 PMC2832260

[B48] Zhang D. Cheng H. Geng L. Kan G. Cui S. Meng Q. (2009). Detection of quantitative trait loci for phosphorus deficiency tolerance at soybean seedling stage. Euphytica 167, 313–322. 10.1007/s10681-009-9880-0

[B49] Zhang D. Cheng H. Hu Z. Wang H. Kan G. Liu C. (2013). Fine mapping of a major flowering time QTL on soybean chromosome 6 combining linkage and association analysis. Euphytica 191, 23–33. 10.1007/s10681-012-0840-8

[B50] Zhang D. Li H. Wang J. Zhang H. Hu Z. Chu S.. (2016). High-density genetic mapping identifies new major loci for tolerance to low-phosphorus stress in soybean. Front. Plant Sci. 7:372. 10.3389/fpls.2016.00372 27065041 PMC4811872

[B51] Zhang D. Liu C. Cheng H. Kan G. Cui S. Meng Q. (2010). Quantitative trait loci associated with soybean tolerance to low phosphorus stress based on flower and pod abscission. Plant Breed. 129, 243–249. 10.1111/j.1439-0523.2009.01682.x

[B52] Zhang D. Song H. Cheng H. Hao D. Wang H. Kan G.. (2014). The acid phosphatase-encoding gene *GmACP1* contributes to soybean tolerance to low-phosphorus stress. PLoS Genet. 10:e1004061. 10.1371/journal.pgen.1004061 24391523 PMC3879153

[B53] Zhao X. Xu J. Zhao M. Lafitte R. Zhu L. Fu B. (2008). QTLs affecting morph-physiological traits related to drought tolerance detected in overlapping introgression lines of rice (*Oryza sativa* L.). Plant Sci. 174, 618–625. 10.1016/j.plantsci.2008.03.009

