# Supplementary figures and images for: A Genetic Relationship between Phosphorus Efficiency and Photosynthetic Traits in Soybean As Revealed by QTL Analysis Using a High-Density Genetic Map
Source: Front Plant Sci. 2016 Jun 28;7:924. doi: 10.3389/fpls.2016.00924 (PMC4923142; doi:10.3389/fpls.2016.00924)

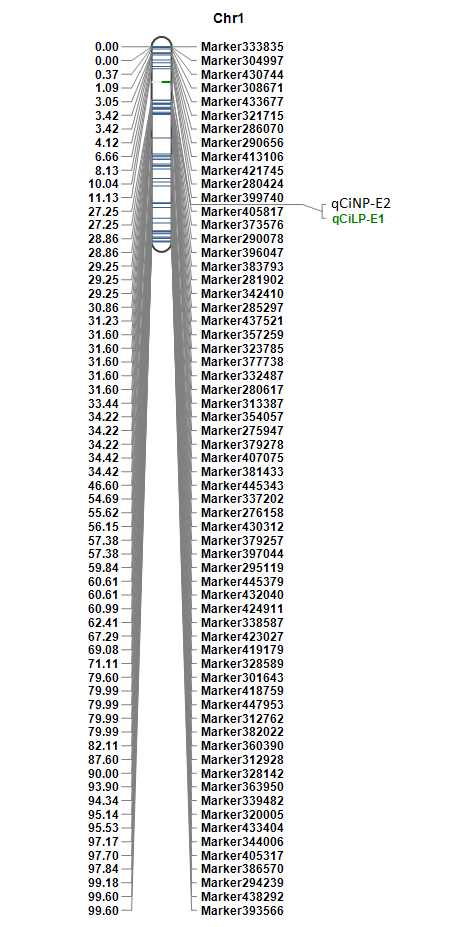

Supplement: Figure S1 — Quantitative trait loci (QTL) for PE and photosynthetic related traits at normal P (NP) and low P (LP) conditions. The designation on the left/right is genetic distance (cM) and marker name. The name of each QTL, is a composite of the influenced trait: RDW, root dry weight; SDW, shoot dry weight; TDW, total dry weight; R/S, root to shoot ratio; PC, P concentration; Pup, phosphorus uptake; PUE, phosphorus use efficiency; BY, biomass yield; PN, net photosynthetic rate; Tr; transpiration rate; and Ci, intercellular carbon dioxide concentration; Co, stomatal conductance; and CC, chlorophyll content; followed by the treatments, environments, and growth stages. See Table 1 for an explanation of trait abbreviations and their units. [file Presentation1.ZIP › Figure S1/Chr.1.tif]

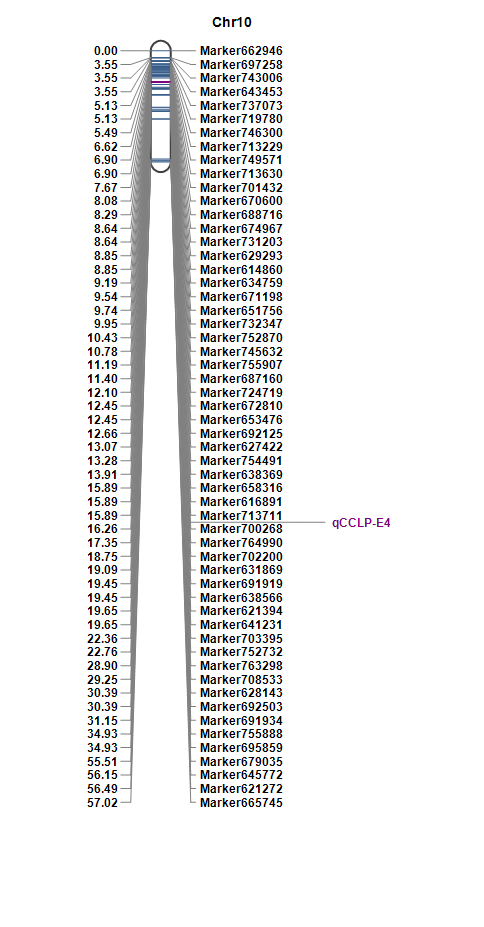

Supplement: Figure S1 — Quantitative trait loci (QTL) for PE and photosynthetic related traits at normal P (NP) and low P (LP) conditions. The designation on the left/right is genetic distance (cM) and marker name. The name of each QTL, is a composite of the influenced trait: RDW, root dry weight; SDW, shoot dry weight; TDW, total dry weight; R/S, root to shoot ratio; PC, P concentration; Pup, phosphorus uptake; PUE, phosphorus use efficiency; BY, biomass yield; PN, net photosynthetic rate; Tr; transpiration rate; and Ci, intercellular carbon dioxide concentration; Co, stomatal conductance; and CC, chlorophyll content; followed by the treatments, environments, and growth stages. See Table 1 for an explanation of trait abbreviations and their units. [file Presentation1.ZIP › Figure S1/Chr.10.tif]

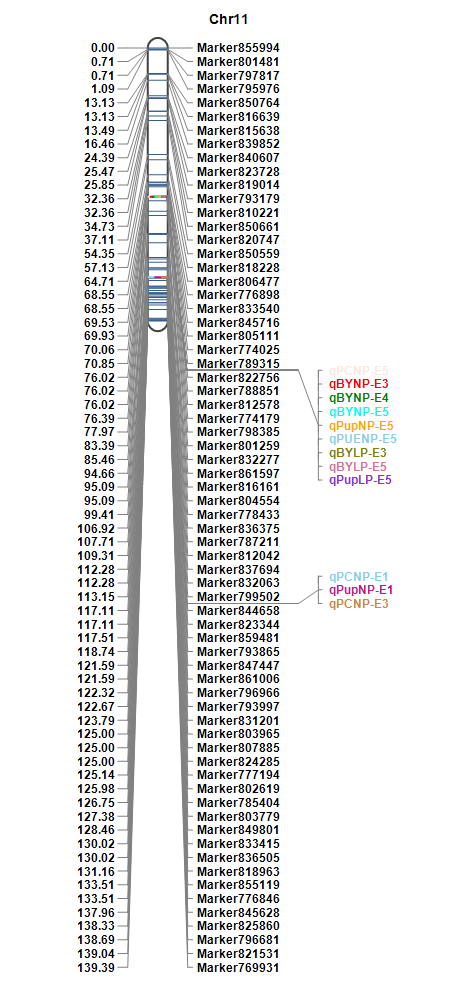

Supplement: Figure S1 — Quantitative trait loci (QTL) for PE and photosynthetic related traits at normal P (NP) and low P (LP) conditions. The designation on the left/right is genetic distance (cM) and marker name. The name of each QTL, is a composite of the influenced trait: RDW, root dry weight; SDW, shoot dry weight; TDW, total dry weight; R/S, root to shoot ratio; PC, P concentration; Pup, phosphorus uptake; PUE, phosphorus use efficiency; BY, biomass yield; PN, net photosynthetic rate; Tr; transpiration rate; and Ci, intercellular carbon dioxide concentration; Co, stomatal conductance; and CC, chlorophyll content; followed by the treatments, environments, and growth stages. See Table 1 for an explanation of trait abbreviations and their units. [file Presentation1.ZIP › Figure S1/Chr.11.tif]

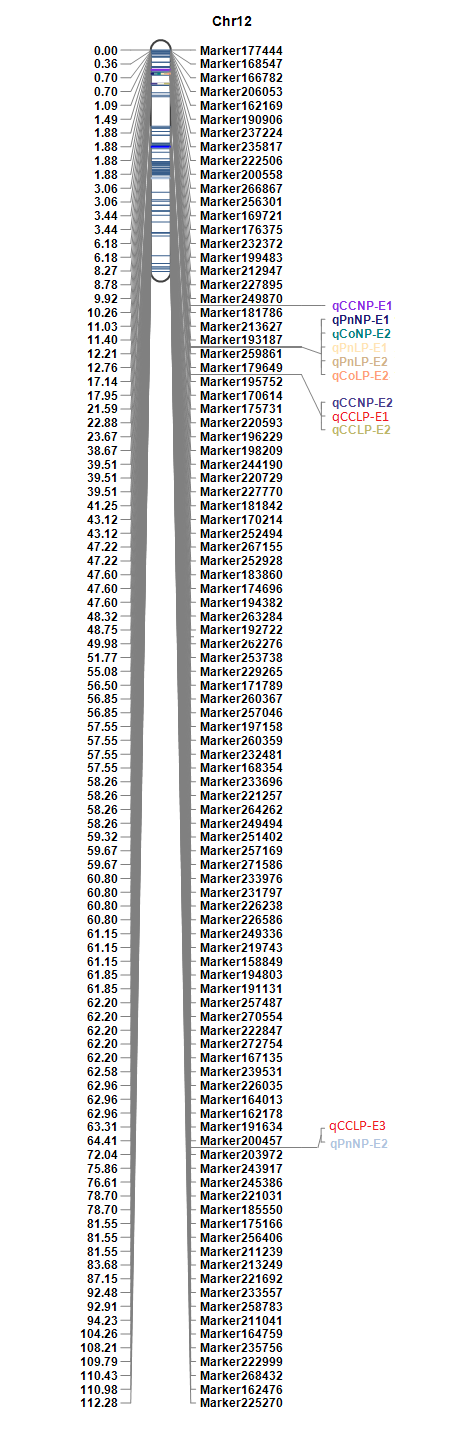

Supplement: Figure S1 — Quantitative trait loci (QTL) for PE and photosynthetic related traits at normal P (NP) and low P (LP) conditions. The designation on the left/right is genetic distance (cM) and marker name. The name of each QTL, is a composite of the influenced trait: RDW, root dry weight; SDW, shoot dry weight; TDW, total dry weight; R/S, root to shoot ratio; PC, P concentration; Pup, phosphorus uptake; PUE, phosphorus use efficiency; BY, biomass yield; PN, net photosynthetic rate; Tr; transpiration rate; and Ci, intercellular carbon dioxide concentration; Co, stomatal conductance; and CC, chlorophyll content; followed by the treatments, environments, and growth stages. See Table 1 for an explanation of trait abbreviations and their units. [file Presentation1.ZIP › Figure S1/Chr.12.tif]

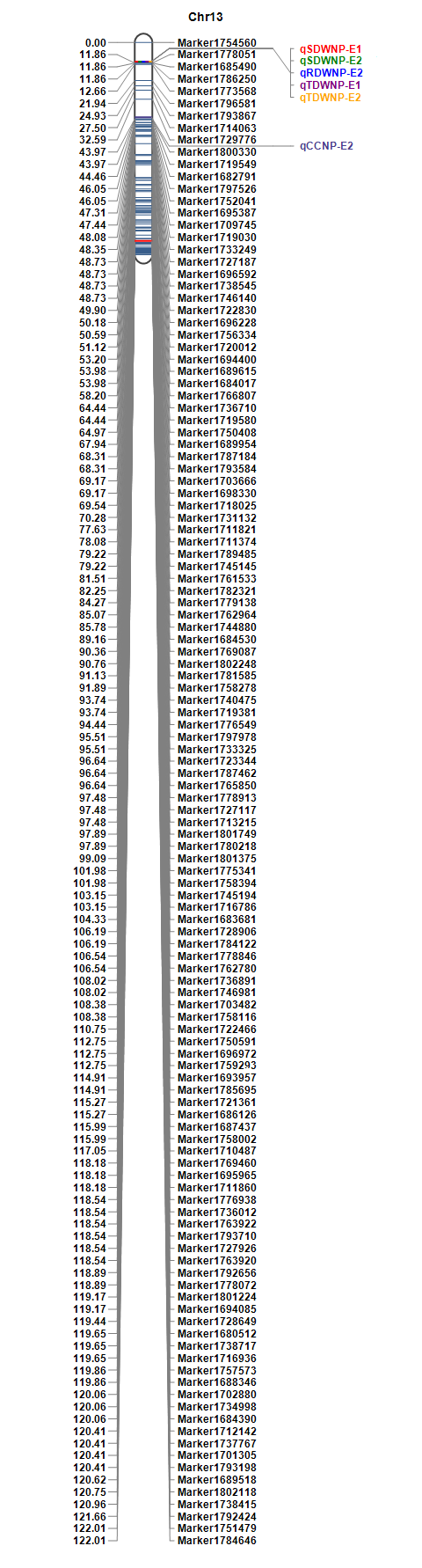

Supplement: Figure S1 — Quantitative trait loci (QTL) for PE and photosynthetic related traits at normal P (NP) and low P (LP) conditions. The designation on the left/right is genetic distance (cM) and marker name. The name of each QTL, is a composite of the influenced trait: RDW, root dry weight; SDW, shoot dry weight; TDW, total dry weight; R/S, root to shoot ratio; PC, P concentration; Pup, phosphorus uptake; PUE, phosphorus use efficiency; BY, biomass yield; PN, net photosynthetic rate; Tr; transpiration rate; and Ci, intercellular carbon dioxide concentration; Co, stomatal conductance; and CC, chlorophyll content; followed by the treatments, environments, and growth stages. See Table 1 for an explanation of trait abbreviations and their units. [file Presentation1.ZIP › Figure S1/Chr.13.tif]

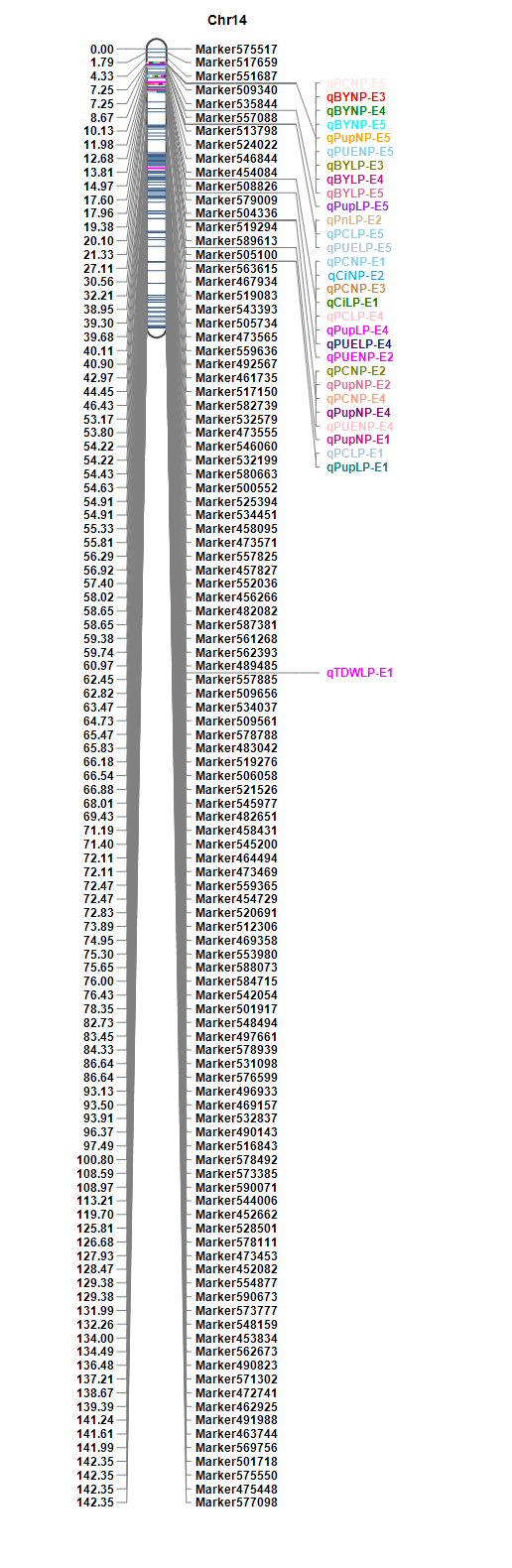

Supplement: Figure S1 — Quantitative trait loci (QTL) for PE and photosynthetic related traits at normal P (NP) and low P (LP) conditions. The designation on the left/right is genetic distance (cM) and marker name. The name of each QTL, is a composite of the influenced trait: RDW, root dry weight; SDW, shoot dry weight; TDW, total dry weight; R/S, root to shoot ratio; PC, P concentration; Pup, phosphorus uptake; PUE, phosphorus use efficiency; BY, biomass yield; PN, net photosynthetic rate; Tr; transpiration rate; and Ci, intercellular carbon dioxide concentration; Co, stomatal conductance; and CC, chlorophyll content; followed by the treatments, environments, and growth stages. See Table 1 for an explanation of trait abbreviations and their units. [file Presentation1.ZIP › Figure S1/Chr.14.tif]

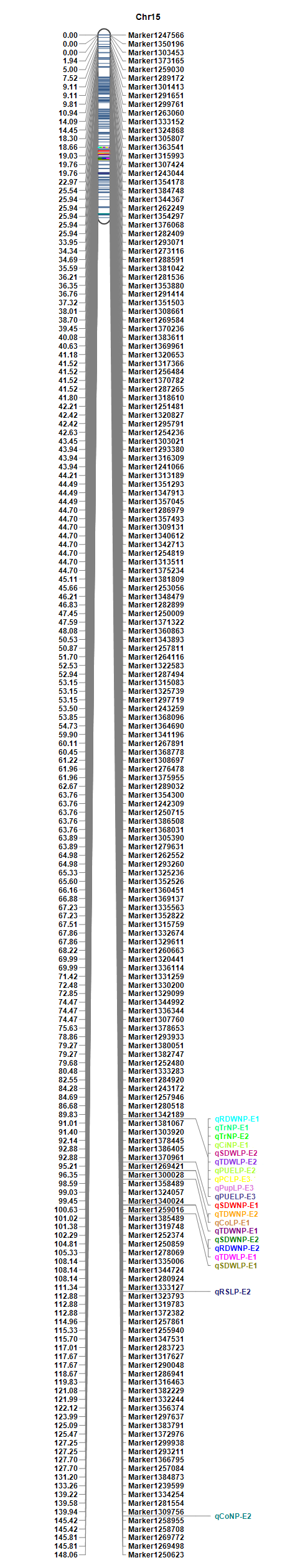

Supplement: Figure S1 — Quantitative trait loci (QTL) for PE and photosynthetic related traits at normal P (NP) and low P (LP) conditions. The designation on the left/right is genetic distance (cM) and marker name. The name of each QTL, is a composite of the influenced trait: RDW, root dry weight; SDW, shoot dry weight; TDW, total dry weight; R/S, root to shoot ratio; PC, P concentration; Pup, phosphorus uptake; PUE, phosphorus use efficiency; BY, biomass yield; PN, net photosynthetic rate; Tr; transpiration rate; and Ci, intercellular carbon dioxide concentration; Co, stomatal conductance; and CC, chlorophyll content; followed by the treatments, environments, and growth stages. See Table 1 for an explanation of trait abbreviations and their units. [file Presentation1.ZIP › Figure S1/Chr.15.tif]

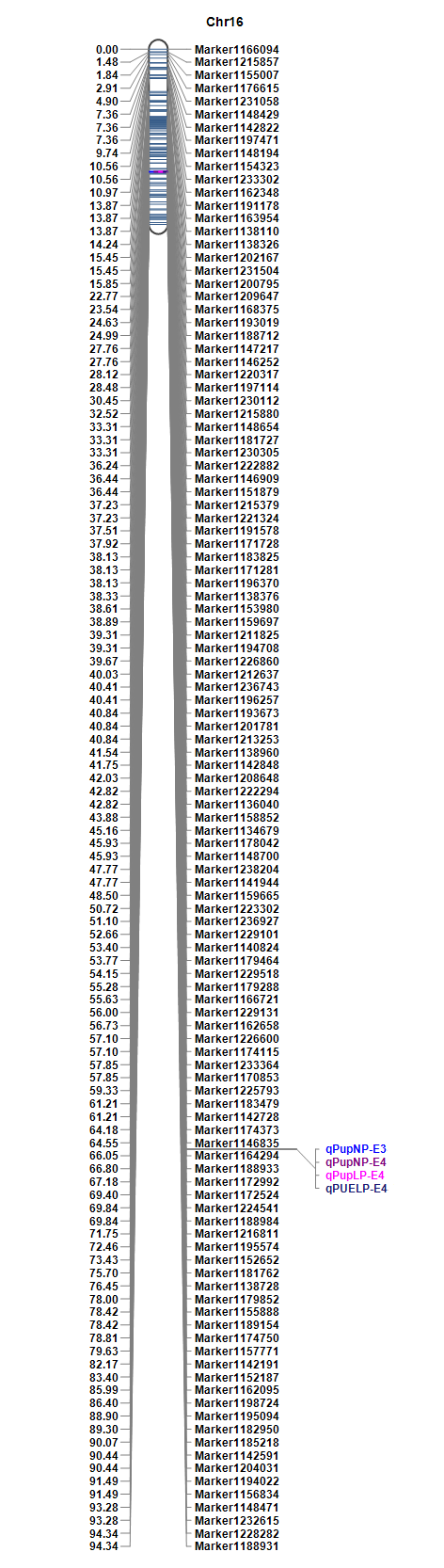

Supplement: Figure S1 — Quantitative trait loci (QTL) for PE and photosynthetic related traits at normal P (NP) and low P (LP) conditions. The designation on the left/right is genetic distance (cM) and marker name. The name of each QTL, is a composite of the influenced trait: RDW, root dry weight; SDW, shoot dry weight; TDW, total dry weight; R/S, root to shoot ratio; PC, P concentration; Pup, phosphorus uptake; PUE, phosphorus use efficiency; BY, biomass yield; PN, net photosynthetic rate; Tr; transpiration rate; and Ci, intercellular carbon dioxide concentration; Co, stomatal conductance; and CC, chlorophyll content; followed by the treatments, environments, and growth stages. See Table 1 for an explanation of trait abbreviations and their units. [file Presentation1.ZIP › Figure S1/Chr.16.tif]

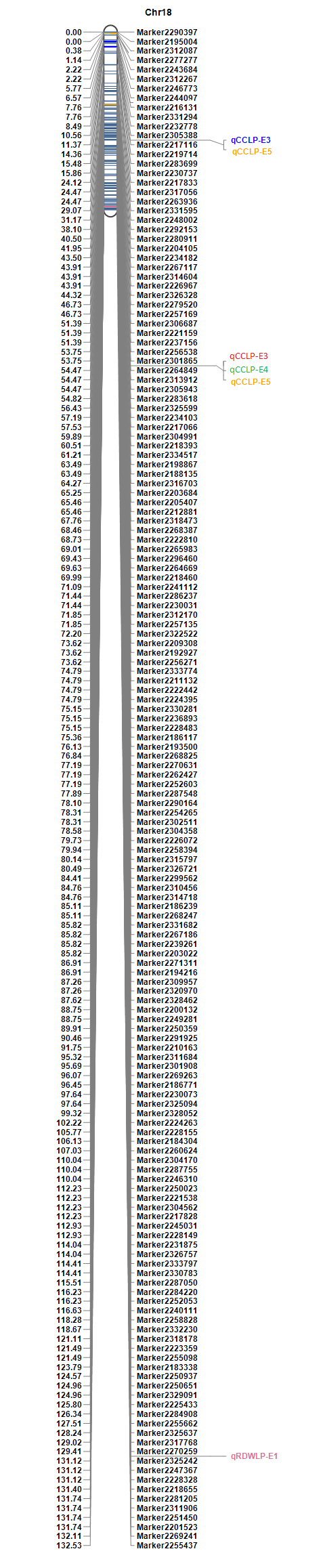

Supplement: Figure S1 — Quantitative trait loci (QTL) for PE and photosynthetic related traits at normal P (NP) and low P (LP) conditions. The designation on the left/right is genetic distance (cM) and marker name. The name of each QTL, is a composite of the influenced trait: RDW, root dry weight; SDW, shoot dry weight; TDW, total dry weight; R/S, root to shoot ratio; PC, P concentration; Pup, phosphorus uptake; PUE, phosphorus use efficiency; BY, biomass yield; PN, net photosynthetic rate; Tr; transpiration rate; and Ci, intercellular carbon dioxide concentration; Co, stomatal conductance; and CC, chlorophyll content; followed by the treatments, environments, and growth stages. See Table 1 for an explanation of trait abbreviations and their units. [file Presentation1.ZIP › Figure S1/Chr.18.tif]

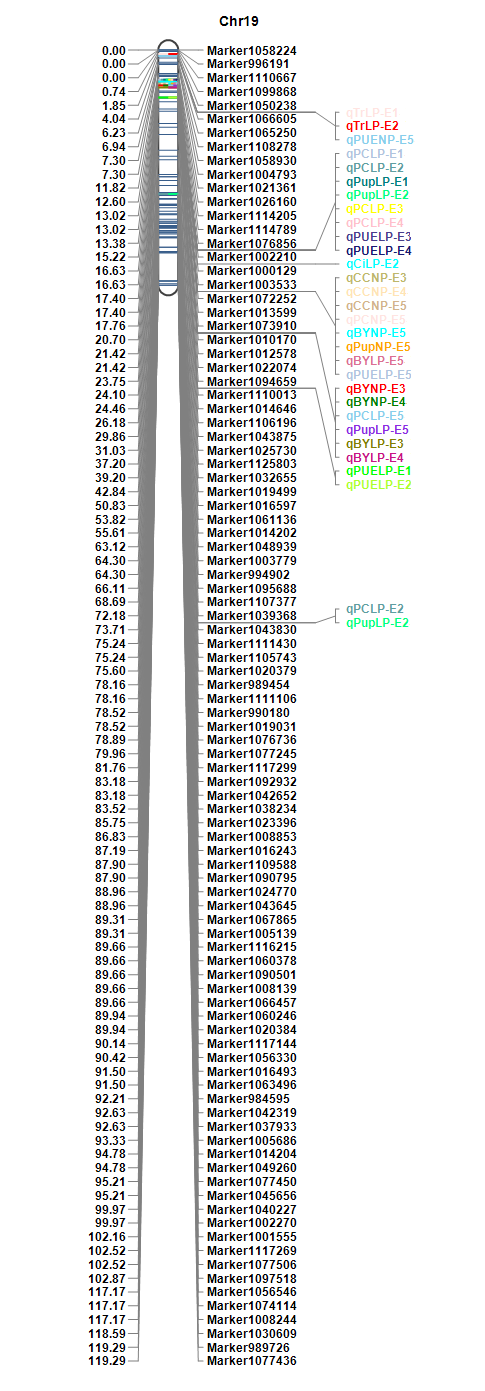

Supplement: Figure S1 — Quantitative trait loci (QTL) for PE and photosynthetic related traits at normal P (NP) and low P (LP) conditions. The designation on the left/right is genetic distance (cM) and marker name. The name of each QTL, is a composite of the influenced trait: RDW, root dry weight; SDW, shoot dry weight; TDW, total dry weight; R/S, root to shoot ratio; PC, P concentration; Pup, phosphorus uptake; PUE, phosphorus use efficiency; BY, biomass yield; PN, net photosynthetic rate; Tr; transpiration rate; and Ci, intercellular carbon dioxide concentration; Co, stomatal conductance; and CC, chlorophyll content; followed by the treatments, environments, and growth stages. See Table 1 for an explanation of trait abbreviations and their units. [file Presentation1.ZIP › Figure S1/Chr.19.tif]

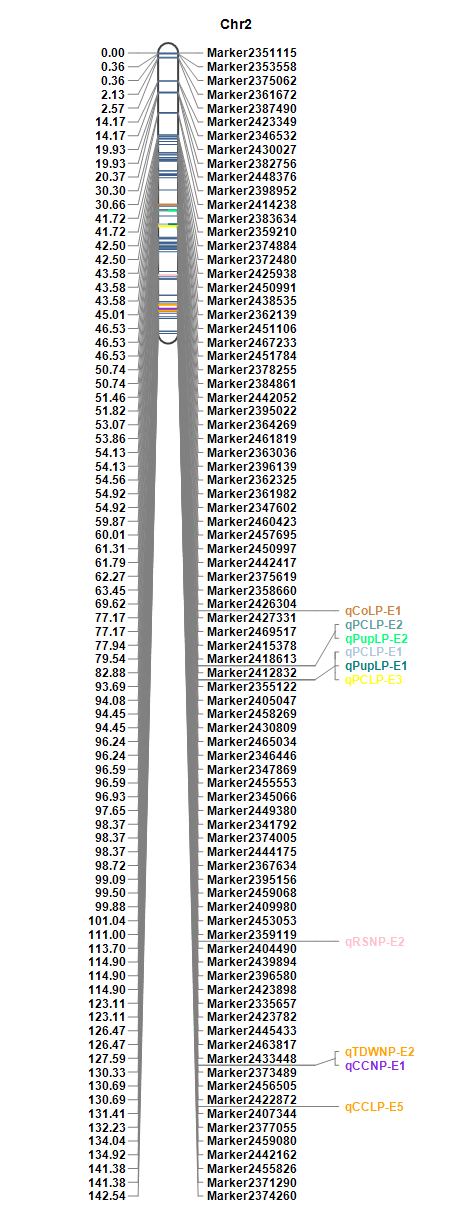

Supplement: Figure S1 — Quantitative trait loci (QTL) for PE and photosynthetic related traits at normal P (NP) and low P (LP) conditions. The designation on the left/right is genetic distance (cM) and marker name. The name of each QTL, is a composite of the influenced trait: RDW, root dry weight; SDW, shoot dry weight; TDW, total dry weight; R/S, root to shoot ratio; PC, P concentration; Pup, phosphorus uptake; PUE, phosphorus use efficiency; BY, biomass yield; PN, net photosynthetic rate; Tr; transpiration rate; and Ci, intercellular carbon dioxide concentration; Co, stomatal conductance; and CC, chlorophyll content; followed by the treatments, environments, and growth stages. See Table 1 for an explanation of trait abbreviations and their units. [file Presentation1.ZIP › Figure S1/Chr.2.tif]

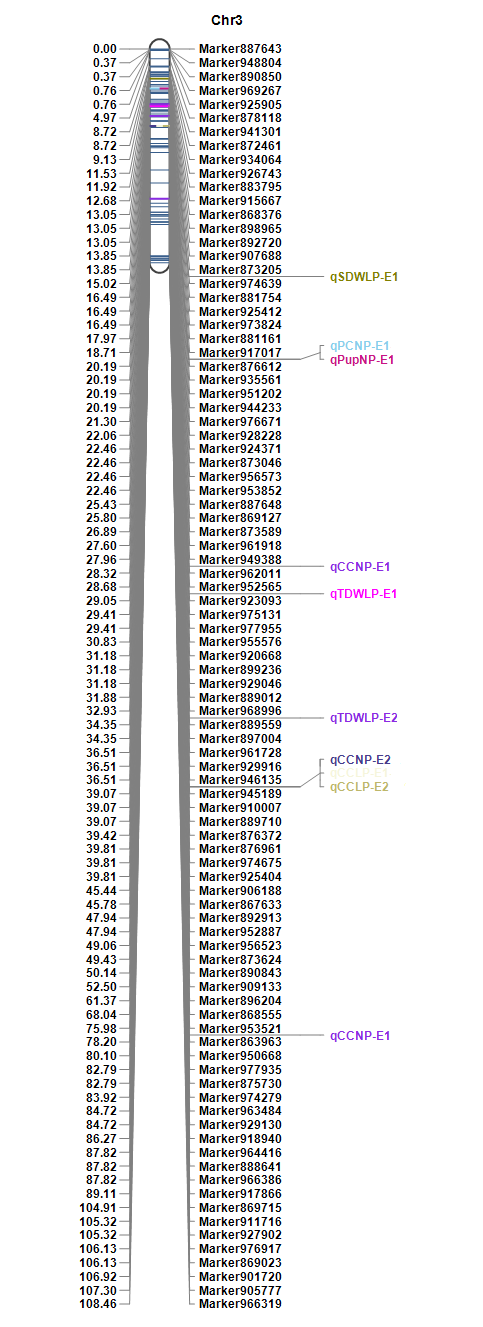

Supplement: Figure S1 — Quantitative trait loci (QTL) for PE and photosynthetic related traits at normal P (NP) and low P (LP) conditions. The designation on the left/right is genetic distance (cM) and marker name. The name of each QTL, is a composite of the influenced trait: RDW, root dry weight; SDW, shoot dry weight; TDW, total dry weight; R/S, root to shoot ratio; PC, P concentration; Pup, phosphorus uptake; PUE, phosphorus use efficiency; BY, biomass yield; PN, net photosynthetic rate; Tr; transpiration rate; and Ci, intercellular carbon dioxide concentration; Co, stomatal conductance; and CC, chlorophyll content; followed by the treatments, environments, and growth stages. See Table 1 for an explanation of trait abbreviations and their units. [file Presentation1.ZIP › Figure S1/Chr.3.tif]

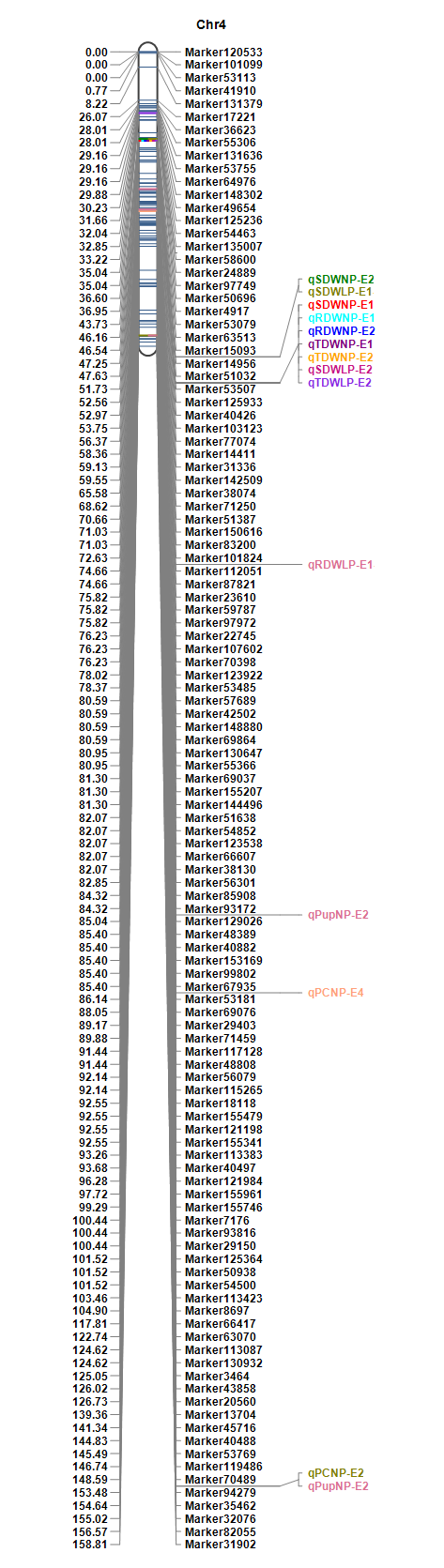

Supplement: Figure S1 — Quantitative trait loci (QTL) for PE and photosynthetic related traits at normal P (NP) and low P (LP) conditions. The designation on the left/right is genetic distance (cM) and marker name. The name of each QTL, is a composite of the influenced trait: RDW, root dry weight; SDW, shoot dry weight; TDW, total dry weight; R/S, root to shoot ratio; PC, P concentration; Pup, phosphorus uptake; PUE, phosphorus use efficiency; BY, biomass yield; PN, net photosynthetic rate; Tr; transpiration rate; and Ci, intercellular carbon dioxide concentration; Co, stomatal conductance; and CC, chlorophyll content; followed by the treatments, environments, and growth stages. See Table 1 for an explanation of trait abbreviations and their units. [file Presentation1.ZIP › Figure S1/Chr.4.tif]

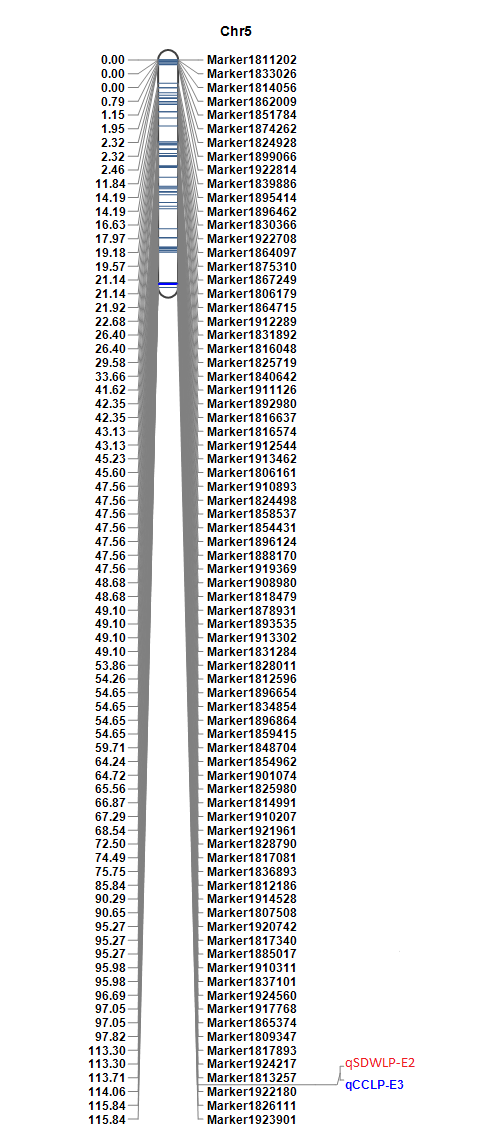

Supplement: Figure S1 — Quantitative trait loci (QTL) for PE and photosynthetic related traits at normal P (NP) and low P (LP) conditions. The designation on the left/right is genetic distance (cM) and marker name. The name of each QTL, is a composite of the influenced trait: RDW, root dry weight; SDW, shoot dry weight; TDW, total dry weight; R/S, root to shoot ratio; PC, P concentration; Pup, phosphorus uptake; PUE, phosphorus use efficiency; BY, biomass yield; PN, net photosynthetic rate; Tr; transpiration rate; and Ci, intercellular carbon dioxide concentration; Co, stomatal conductance; and CC, chlorophyll content; followed by the treatments, environments, and growth stages. See Table 1 for an explanation of trait abbreviations and their units. [file Presentation1.ZIP › Figure S1/Chr.5.tif]

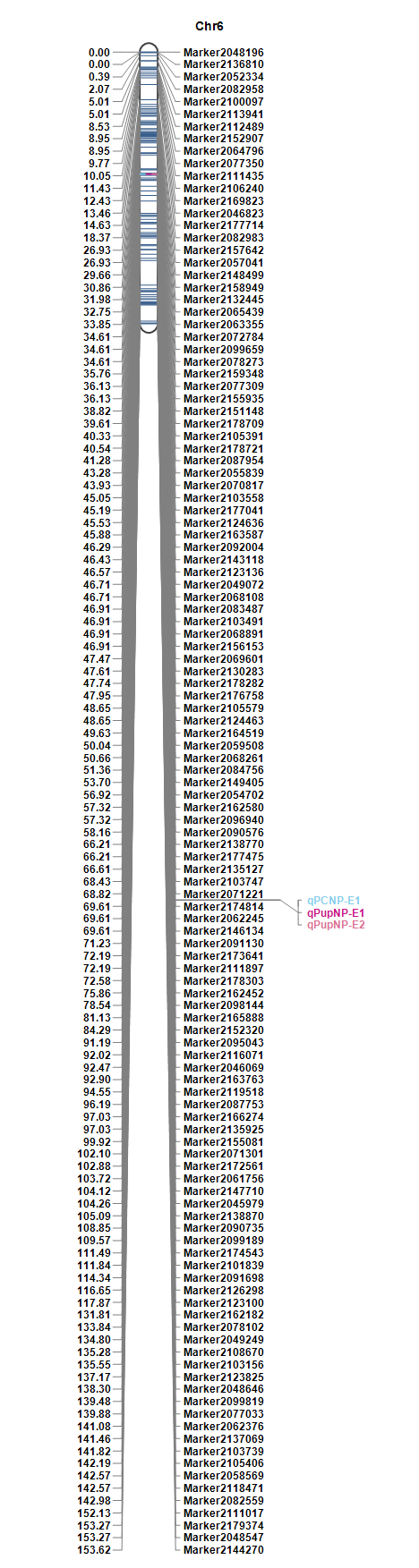

Supplement: Figure S1 — Quantitative trait loci (QTL) for PE and photosynthetic related traits at normal P (NP) and low P (LP) conditions. The designation on the left/right is genetic distance (cM) and marker name. The name of each QTL, is a composite of the influenced trait: RDW, root dry weight; SDW, shoot dry weight; TDW, total dry weight; R/S, root to shoot ratio; PC, P concentration; Pup, phosphorus uptake; PUE, phosphorus use efficiency; BY, biomass yield; PN, net photosynthetic rate; Tr; transpiration rate; and Ci, intercellular carbon dioxide concentration; Co, stomatal conductance; and CC, chlorophyll content; followed by the treatments, environments, and growth stages. See Table 1 for an explanation of trait abbreviations and their units. [file Presentation1.ZIP › Figure S1/Chr.6.tif]

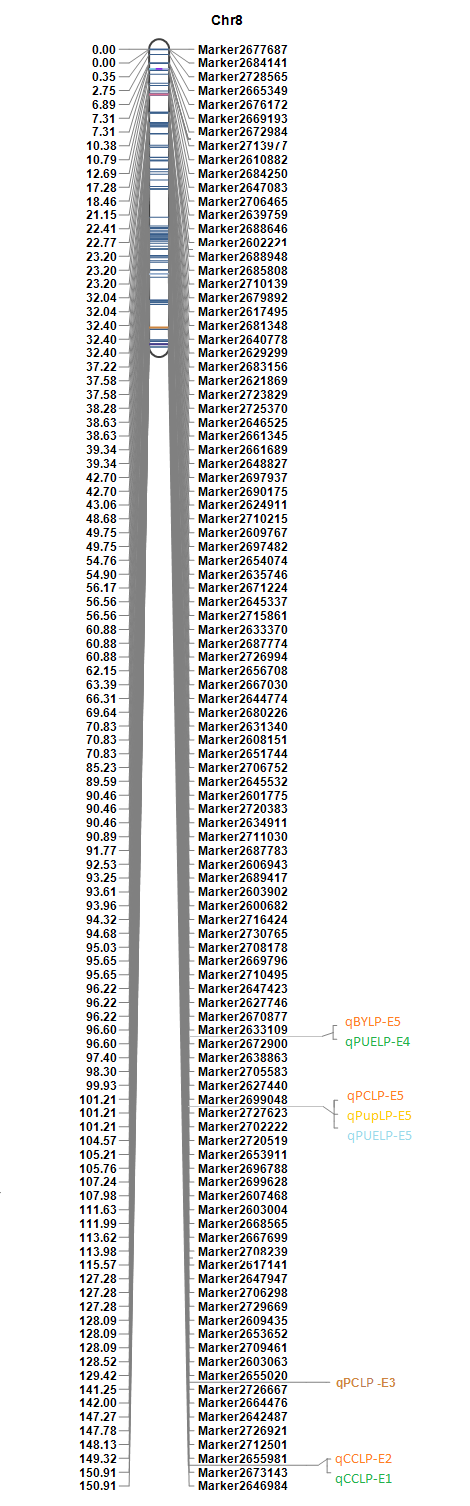

Supplement: Figure S1 — Quantitative trait loci (QTL) for PE and photosynthetic related traits at normal P (NP) and low P (LP) conditions. The designation on the left/right is genetic distance (cM) and marker name. The name of each QTL, is a composite of the influenced trait: RDW, root dry weight; SDW, shoot dry weight; TDW, total dry weight; R/S, root to shoot ratio; PC, P concentration; Pup, phosphorus uptake; PUE, phosphorus use efficiency; BY, biomass yield; PN, net photosynthetic rate; Tr; transpiration rate; and Ci, intercellular carbon dioxide concentration; Co, stomatal conductance; and CC, chlorophyll content; followed by the treatments, environments, and growth stages. See Table 1 for an explanation of trait abbreviations and their units. [file Presentation1.ZIP › Figure S1/Chr.8.tif]

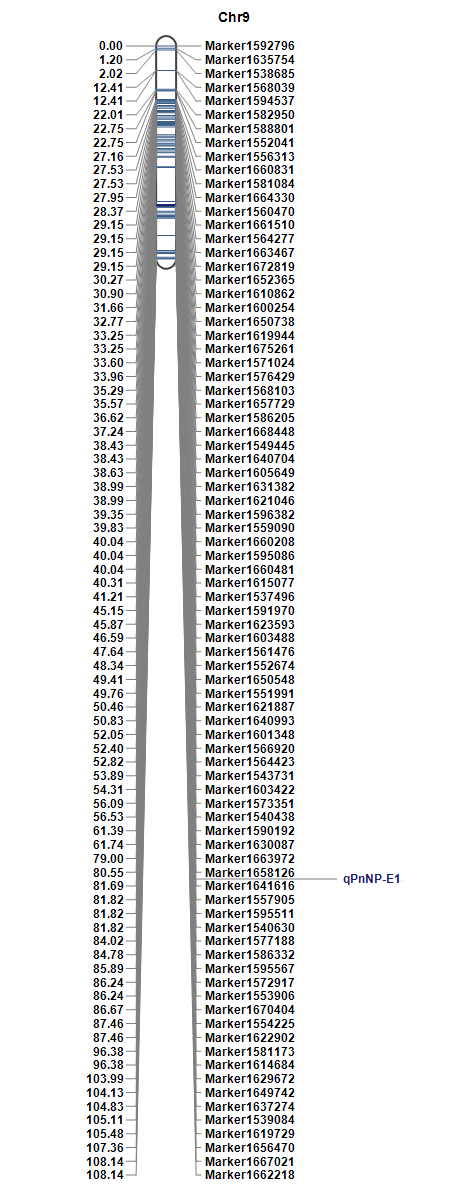

Supplement: Figure S1 — Quantitative trait loci (QTL) for PE and photosynthetic related traits at normal P (NP) and low P (LP) conditions. The designation on the left/right is genetic distance (cM) and marker name. The name of each QTL, is a composite of the influenced trait: RDW, root dry weight; SDW, shoot dry weight; TDW, total dry weight; R/S, root to shoot ratio; PC, P concentration; Pup, phosphorus uptake; PUE, phosphorus use efficiency; BY, biomass yield; PN, net photosynthetic rate; Tr; transpiration rate; and Ci, intercellular carbon dioxide concentration; Co, stomatal conductance; and CC, chlorophyll content; followed by the treatments, environments, and growth stages. See Table 1 for an explanation of trait abbreviations and their units. [file Presentation1.ZIP › Figure S1/Chr.9.tif]

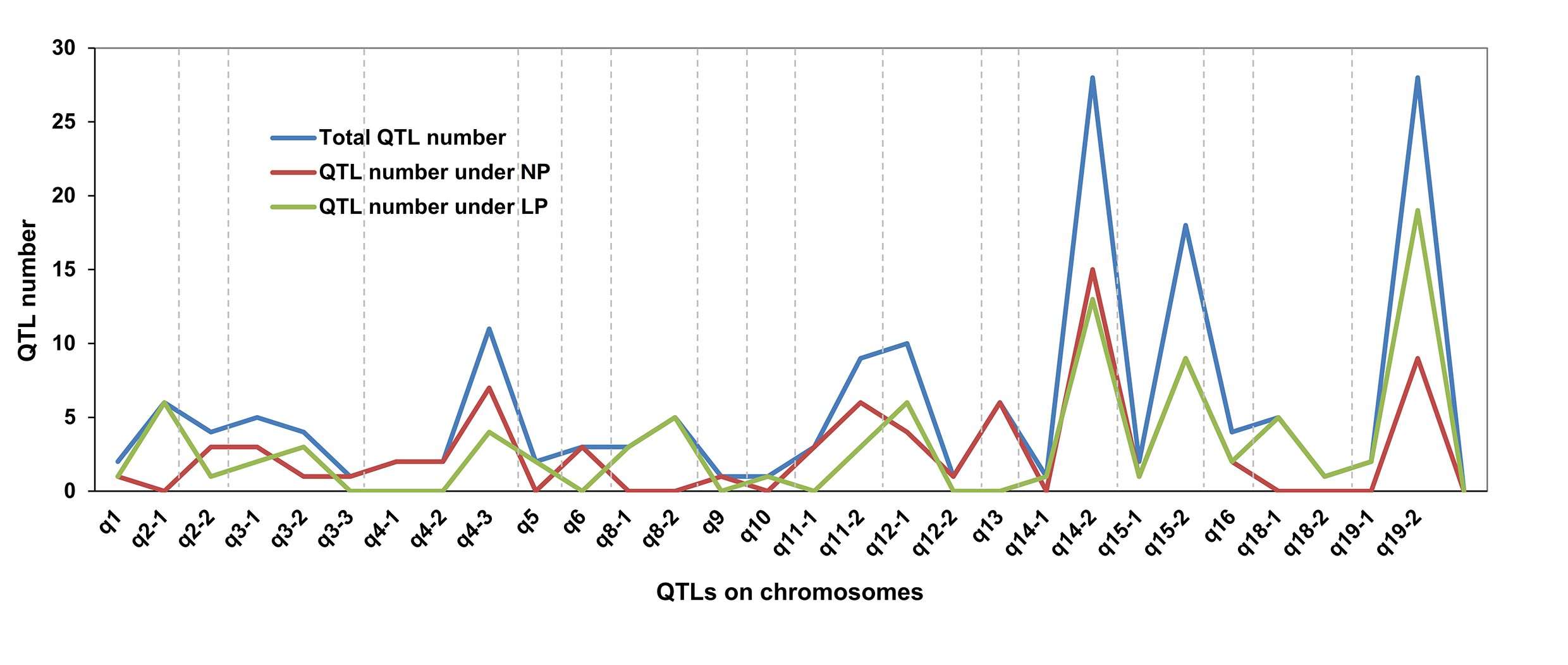

Supplement: Figure S2 — QTLs indicated by the number of significant QTLs plotted for 20 chromosomes (Chr.01-Chr.20) under normal P (NP) and low P (LP) conditions. The gray dotted vertical lines separate the 20 chromosomes. [file Image1.TIF]
